# Statistical Thermodynamics of Chiral Skyrmions in a Ferromagnetic Material

**DOI:** 10.3390/ma12223702

**Published:** 2019-11-09

**Authors:** Roberto Zivieri

**Affiliations:** Department of Mathematical and Computer Sciences, Physical Sciences and Earth Sciences, University of Messina, Contrada di Dio, 98166 Messina, Italy; zivieri@fe.infn.it; Tel.: +39-0532-974232

**Keywords:** solitons, topological defects, chiral magnetic skyrmions, Dzyaloshinskii–Moriya interaction, microcanonical ensemble, equilibrium statistical mechanics of topological defects, skyrmion free energy and partition function, pressure and equation of state

## Abstract

Solitons are a challenging topic in condensed matter physics and materials science because of the interplay between their topological and physical properties and for the crucial role they play in topological phase transitions. Among them, chiral skyrmions hosted in ferromagnetic systems are axisymmetric solitonic states attracting a lot of attention for their dazzling physical properties and technological applications. In this paper, the equilibrium statistical thermodynamics of chiral magnetic skyrmions developing in a ferromagnetic material having the shape of an ultrathin cylindrical dot is investigated. This is accomplished by determining via analytical calculations for both Néel and Bloch skyrmions: (1) the internal energy of a single chiral skyrmion; (2) the partition function; (3) the free energy; (4) the pressure; and (5) the equation of state of a skyrmion diameters population. To calculate the thermodynamic functions for points (2)–(5), the derivation of the average internal energy and of the configurational entropy is crucial. Numerical calculations of the thermodynamic functions for points (1)–(5) are applied to Néel skyrmions. These results could advance the field of materials science with special regard to low-dimensional magnetic systems.

## 1. Introduction

The thermodynamic description of topological defects and topological phase transitions has been one of most important challenges of the modern condensed matter physics. The leading works on the thermodynamics of topological defects and the relevant underlying physics are the ones dating back to the 70s of Berezinskii and of Kosterlitz and Thouless on defect-mediated phase transitions in two-dimensional (2D) XY superfluid model. In particular, Berezinskii studied the low-temperature state of one-dimensional (1D) and 2D classical and quantum systems such as crystals, isotropic magnetic substances, and superfluids and superconductors having continuous symmetry, showing that in both kinds of systems the long-range order is destroyed due to the increasing fluctuations of the ordering parameter with increasing size [1,2]. Kosterlitz and Thouless deepened this type of investigation, arguing the existence of topological defects having the form of vortices in physical systems described by the XY model, such as superfluids [3,4,5]. They studied the thermodynamic behavior of these systems by means of the calculation of the Helmholtz free energy *F* showing that, for *T*→0 K and with increasing size, *F* can be minimized if no vortices appear, while above a critical temperature *F* is minimized if there is the formation of couples of unpaired vortices and anti-vortices. On the basis of this analysis, they introduced the concept of defect-mediated topological phase transition in condensed matter physics systems, the so-called infinite-order Berezinskii–Kosterlitz–Thouless phase transition.

After these seminal works, a great deal of attention has been paid to study the interplay between topology and physics characterizing solitons in condensed matter systems and its relevant implications. Skyrmions are topologically protected objects, characteristic of certain classes of nonlinear continuous models, that could be present in various condensed matter systems such as, for instance, Bose–Einstein condensates [6], chiral nematic liquids [7], superconductors [8], superfluids [9]. Their name was first proposed as topological solitons field-like solutions for pions in nuclear physics whose topological stability was determined by the conservation of their baryon number [10].

The interest has grown in the last years in the field of magnetic materials after the discovery of magnetic skyrmions, the smallest magnetic structures in low-dimensional magnetic systems behaving like particles, that are topologically stable configurations arising in ferromagnetic samples. Magnetic skyrmions are characterized by a skyrmion number $S=1/\left(4\pi \right)\int d^{2}\rho \;m\cdot \left(\frac{\partial m}{\partial x}\times \frac{\partial m}{\partial y}\right)$ where **m**(***ρ***) = **M**(***ρ***)/*M*_s_ is the unit magnetization vector with **M** the magnetization, ***ρ*** = (*x*,*y*), and *M*_s_ the saturation magnetization, and ∂/∂*x* and ∂/∂*y* are first partial derivatives. During the last decade, the classical modelization of single chiral magnetic skyrmions and their observation has attracted a lot of physicists and materials science researchers for the new physics that characterizes this type of solitons, their dazzling properties, and the several technological applications. Specifically, they can be employed as information carriers in logic and spintronic devices because very low spin currents are needed to move them and therefore they contribute to the advancing of the field of skyrmionics [11,12]. The key energy interaction that stabilizes chiral Néel (hedgehog-like) and Bloch (vortex-like) skyrmions determining the sense of rotation of the magnetization is the Dzyaloshinskii–Moriya interaction (DMI). This interaction was discovered developing a thermodynamic theory of “weak” ferromagnetism of antiferromagnetic crystals resulting in an anti-symmetric spin coupling [13], and by generalizing the Anderson theory of superexchange with the inclusion of the spin-orbit interaction that gives rise to anisotropic effects in “weak” ferromagnets [14]. The DMI can be regarded as a relativistic form of chiral exchange energy due to the lack or breaking of inversion symmetry in bulk crystalline systems such as B20 cubic crystal structures (MnSi, FeGe) [12]. The DMI may lead to the spontaneous formation of ground skyrmion states in condensed matter systems with chiral interactions, to the spontaneous formation of 2D lattice lines observed in a chiral magnet such as MnSi by means of neutron scattering and Hall effect measurements [15,16,17,18]. The DMI may play a crucial role also at the interfaces in magnetic Ir|Co|Pt multilayers hosting room temperature magnetic skyrmions [19] or in *β*-type CuZnMn alloys where skyrmion lattices above room temperature were observed [20]. Its microscopic origin at the film interface was recently investigated [21]. The first theoretical prediction and description of chiral magnetic skyrmions in thin magnetic films was done by Bogdanov and Rössler [22]. This prediction was confirmed a few years ago by the observation of a spontaneous atomic-scale magnetic skyrmion lattice in two dimensions by Heinze et al. [23]. In particular, the bulk DMI interaction typical of *D*_2d_ systems stabilizes chiral Bloch skyrmions [24], while the interfacial DMI (IDMI) stabilizes chiral Néel skyrmions [12]. The tilting of edge moments in nanostructures hosting skyrmions [25], the evolution of single chiral skyrmions under an applied magnetic field [26], the current induced motion of different types of chiral skyrmions [27,28,29,30], the current-induced rotational torques in skyrmion lattices [31], and the current-dependent skyrmion Hall angle [32] were also studied. In addition, the breathing dynamics [33] and the skyrmion state stability [34,35] were also investigated.

Recently, attention was also paid to study thermal properties of magnetic skyrmions, with special regard to the investigation of skyrmion entropy. In particular, an estimation of the skyrmion entropy has been made from experimental data, and in bulk B20 compounds the concept of skyrmion entropy has been used to classify the magnetization phase transition at the transition temperature [36,37]. In addition, the skyrmion entropy has been included in the Arrhenius law in order to explain the disagreement between the measurement and the calculation of the lifetime of a skyrmion lattice [38].

In a recent paper, a theoretical model was developed to study magnetic skyrmions thermodynamics computing by means of a classical approach to the configurational entropy at equilibrium based on the analogy between a skyrmion diameters population referred to one skyrmion and the Maxwell–Boltzmann statistics of the ideal gas [39] using scaling magnetic parameters as a function of temperature *T* [35]. The entropy *S* = *S*(*T*) of a three-dimensional (3D) Néel magnetic skyrmion diameters population was calculated as an expectation value in a way similar to Shannon’s information entropy. Because of this definition, the skyrmion diameter dependent Helmholtz free energy, *F*(*D*_sky_) = *E* − *T S* (with *E* = *E*(*D*_sky_) the skyrmion energy and *D*_sky_ the skyrmion diameter), also exhibits a square diameter dependence for each *T* and the mean square fluctuation of energy of the skyrmion diameters around its average value population is equivalent to the mean square fluctuation of free energy *F*(*D*_sky_) around its average value.

Recently, in some excellent papers the skyrmion energy has been analytically calculated using either a 1D distribution [40] that was used to describe properties of thick-walled magnetic domains in uniaxial platelets [41] or a two-dimensional domain distributions [25,42], or numerically calculated using an accurate 2D magnetization distribution recently proposed to describe the magnetization texture, namely the orientation of the magnetization vector in the *xy* plane and with respect to the *z* direction, of the Néel (hedgehog) chiral skyrmion [35,43]. Additionally, in [42] the magnetostatic contribution of the volume charges has also been included in the skyrmion energy computation.

However, a systematic theoretical study describing the classical statistical thermodynamic behavior of chiral magnetic skyrmions at equilibrium is still lacking in the literature. In this work, a statistical thermodynamic description of chiral magnetic skyrmions that are hosted in ultrathin cylindrical dots based on a classical approach is given, strengthening the analogy with the ideal gas. This is accomplished via the following key results obtained by means of the analytical and numerical calculation of the following physical quantities at a given *T*:

(1) the calculation of the skyrmion energy for both Néel and Bloch skyrmions that is regarded as the internal energy;
(2) the determination of the partition function and of the free energy of a skyrmion diameters population within a microcanonical ensemble;
(3) the derivation of the skyrmion pressure from the free energy of a skyrmion diameters population and of an equation of state linking the thermodynamic variables *P*, *V*, and *T*.

In addition, it is shown that the configurational entropy calculated for a Nèel skyrmion diameters population takes the same form for a Bloch skyrmions population. This feature also characterizes the partition function and the pressure and results from the fact that the skyrmion internal energy has a quadratic dependence on the skyrmion diameter (or radius) independently of the topological texture and skyrmion number. Therefore, it can be concluded that the statistical thermodynamics qualitative description of axisymmetric solitonic states does not depend on the skyrmion magnetization texture of the chiral skyrmion under study.

In this study the skyrmion energy is analytically computed starting from the 2D magnetization distribution proposed to describe the magnetization texture of the Néel (hedgehog) chiral skyrmion [35,43]. This accurate 2D magnetization distribution recovers, in the isotropic case and for dominating exchange interaction, the Belavin–Polyakov soliton [44] solution and can be easily extended to describe the magnetization texture of the Bloch (vortex-like) chiral skyrmion. In this respect, here it is shown that, for both magnetization textures, the skyrmion energy can be expressed as a combination of elementary transcendental functions and is actually regarded as an internal energy. Owing to some reasonable approximations, from the skyrmion energy a simple analytical form of the equilibrium skyrmion radius in the region of metastability depending on the scaled magnetic parameters is obtained.

The partition function of a skyrmion diameters population is a key physical quantity to describe their statistical thermodynamics. Indeed, it allows to understand the connection between the occupation of microscopic states by the skyrmions population and the macroscopic thermodynamic variables of state, such as the skyrmion free energy and the entropy.

The definition of a skyrmion pressure for a skyrmion diameters population of average volume <*V*> at a given temperature *T* is accomplished exploiting the analogy with the pressure exerted by the molecules of a gas on the walls of the container. The derivation of the skyrmion pressure *p* also allows writing an equation of state linking the thermodynamic variables pressure *p*, volume *V*, and *T* equivalent to the one for an ideal gas. This general relation is valid for any single skyrmion magnetization texture. It is shown that, for *D*_sky_ = <*D*_sky_>, the equation of state reduces to the well-known one for an ideal gas *pV* = *k*_B_*T* for the number of particles *N* = 1. However, for diameters around the average diameter, unlike the ideal gas characterized by the universal constant *R*, it is not possible to define for a skyrmions population a universal constant.

Calculations (1)–(3) are not only important for understanding the thermodynamic properties of chiral skyrmions that form in ultrathin ferromagnetic dots but could enable a further comprehension of the magnetic properties of ferromagnetic materials hosting these topological objects, suggesting new thermodynamic measurements able to confirm the predictions. Especially, calculations (2) and (3) differentiate the analysis and the aim of this work with respect to that of recent studies of the literature that mainly focus on the dynamical properties of magnetic skyrmions treating the equilibrium statistical properties of magnetic skyrmions in the region of metastability within a microcanonical ensemble.

This paper is organized as follows: in Section 2, Methods, the model is presented. First, the skyrmion energy is analytically calculated for both Néel and Bloch magnetic skyrmions according to a simple variable transformation and to some reasonable approximations. Then, the statistical thermodynamics of a skyrmion diameters population is studied within a microcanonical ensemble via the calculation of the partition function, the Helmholtz free energy, and the pressure and by means of the derivation of an equation of state that is similar to the one for an ideal gas. In Section 3, Results and Discussion, the model outlined in Section 2 is applied to a diameters population of Néel skyrmions and the behavior of the corresponding thermodynamic quantities is discussed. The model is also benchmarked via the comparison of the equilibrium diameters as a function of the external magnetic field with available experimental data taken from the literature. In Section 4 Conclusions are drawn.

## 2. Methods

In the following calculations the polar coordinates in the dot plane ***ρ*** = (*ρ*,*φ*) are introduced, where *ρ* is the radial coordinate and *φ* is the azimuthal coordinate defining spherical angles (*θ*,Φ) of the magnetization vector as functions of ***ρ***. The magnetization unit vector is **m** = **M**/*M*_s_.

It is assumed that: (1) the magnetic skyrmion is hosted in an ultrathin cylindrical dot with thickness of less than 1 nm, allowing to consider the static magnetization uniform (*∂***m**/*∂z* = 0)) along the thickness (*z*-coordinate); (2) the magnetization distribution is circularly symmetric, neglecting deviations from the axial symmetry with respect to the out-of-plane direction (*z*-axis) so that at the equilibrium *θ* = Θ_0_(***ρ***), with $\Theta_{0}\left(\rho \right)$ the equilibrium magnetization distribution and Φ_0_(***ρ***) = *φ +φ*_0_. In a cylindrical reference frame (*ρ*, *ϕ*, *z*), the general magnetization distribution of the magnetic skyrmion takes the form **m** = (sin*θ* cos*ϕ*_0_, sin*θ* sin*ϕ*_0_, cos*θ*) with 0 ≤ *θ* ≤ *π.*

The radial Néel (hedgehog) magnetic skyrmion is characterized by a chirality *χ* = sign(*m_ρ_*) obtained setting either $\varphi_{0}=0$ (outwardly) or $\varphi_{0}=\pi$(inwardly) with *χ* = ±1, respectively and by a magnetization texture **m** = (±sin*θ*, 0, cos*θ*), while the Bloch (vortex-like) magnetic skyrmion is characterized by a chirality *χ* = sign(*m_φ_*) = sin*φ*_0_, obtained setting either *φ*_0_ = π/2 (counter-clockwise) or *φ*_0_ = 3*π*/2 (clockwise) with *χ* = ±1, respectively and by a magnetization texture **m**= (0,±sin*θ*, cos*θ*).

### 2.1. Skyrmion Energy Density

The general expression of the skyrmion energy density as a function of **m** is:

(1) $$\varepsilon =A\;{\left(\nabla m\right)}^{2}+\varepsilon_{\mathrm{DMI}}-K_{u}m_{z}^{2}-\frac{1}{2}\mu_{0}M_{s}m\cdot H_{\mathrm{dem}}-\mu_{0}M_{s}m\cdot H_{\mathrm{ext}}$$

The skyrmion energy density includes the exchange *ε*_exch_, the interfacial DMI (IDMI) *ε*_IDMI_ = *D* (*m_z_*(∇· **m**) − (**m**· ∇)*m_z_*) (Néel skyrmion), or the bulk DMI *ε*_Bulk-DMI_ = *D* (**m** · (∇ × **m**)) (Bloch skyrmion), the perpendicular uniaxial anisotropy *ε*_ani_, the magnetostatic (demagnetization) anisotropy *ε*_dem_, and the Zeeman term *ε*_extfield_, where the subscript “extfield” denotes “external field”. Here, **H**_dem_ = (0,0, *H*_dem_) with *H*_dem_ = −*M*_s_ *m_z_* in the ultrathin approximation so that *ε*_dem_ = ½ *µ*_0_ *M*_s_^2^. In the following subsections, the analytical calculations of every contribution are oulined.

#### 2.1.1. Exchange Energy Density

In this subsection, the ferromagnetic exchange energy density is calculated. In its general form it reads *ε*_exch_ = *A*(∇**m**)^2^, with *A* being the exchange stiffness constant and ${\left(\nabla m\right)}^{2}={\left(\nabla m_{x}\right)}^{2}+{\left(\nabla m_{y}\right)}^{2}+{\left(\nabla m_{z}\right)}^{2}\;\mathrm{with}\;\nabla =\frac{\partial }{\partial \rho }\hat{\rho }+\frac{1}{\rho }\frac{\partial }{\partial \phi }\hat{\phi }+\frac{\partial }{\partial z}\hat{z},\;m_{x}=\pm \cos\phi \sin\theta ,\;m_{y}=\pm \sin\phi \sin\theta \;\mathrm{and}\;m_{z}=\cos\theta \;\mathrm{with}\;\theta =\Theta_{0}\left(\rho \right).$

Replacing the above expressions, one gets $\varepsilon_{\mathrm{exch}}=A\left({\left(\frac{d\theta }{d\rho }\right)}^{2}+\frac{\sin^{2}\theta }{\rho^{2}}\right)$ for both Néel and Bloch skyrmions and *ε*_exch_ does not depend on chirality.

#### 2.1.2. DMI Energy Density

First, the IDMI energy density *ε*_IDMI_ = *D*(*m_z_*(∇· **m**)−(**m**· ∇)*m_z_*) is calculated. In particular, $m_{z}\left(\nabla \cdot m\right)=m_{z}\left(\frac{1}{\rho }\frac{\partial }{\partial \rho }\left(\rho m_{\rho }\right)+\frac{1}{\rho }\frac{\partial m_{\varphi }}{\partial \varphi }+\frac{\partial m_{z}}{\partial z}\right)$ and $\left(m\cdot \nabla \right)m_{z}=\left(m_{\rho }\frac{\partial }{\partial \rho }+m_{\varphi }\frac{1}{\rho }\frac{\partial }{\partial \varphi }+m_{z}\frac{\partial }{\partial z}\right)\;m_{z}$.

One gets $\varepsilon_{\mathrm{IDMI}}=\pm D\left(\frac{\sin\;\theta \;\cos\;\theta }{\rho }+\frac{d\theta }{d\rho }\right)$ for a Néel (hedgehog) skyrmion where the + (−) sign in front of *D* refers to *χ* = +1 or radially outward (*χ* = −1 or radially inward) chirality.

The general form of the bulk DMI energy density is *ε*_Bulk-DMI_ = *D* [**m**·(∇ × **m)**], with **m**·(∇ × **m) =** (*m_ρ_*,*m_ϕ_*,*m_z_*)·**(**1/*ρ* ∂*m_z_/*∂*ϕ* − ∂*m_ϕ_/*∂*m_z_*, ∂*m_ρ_/*∂*z* − ∂*m_z_/*∂*ρ*, 1/*ρ* (∂/∂*ρ* (*ρ m_ϕ_*) −∂*m_ρ_/*∂*ϕ*)). For a Bloch (vortex-like) skyrmion in a thin ferromagnetic dot $\varepsilon_{\mathrm{BulkDMI}}=\pm D\left(\frac{d\theta }{d\rho }+\frac{\sin\theta \cos\theta }{\rho }\right)$, where the + (−) sign in front of *D* refers to *χ* = +1 or counter-clockwise (*χ* = −1 or clockwise) chirality.

Therefore, in the ultrathin film limit, the bulk DMI energy density of a Bloch skyrmion assumes the same expression of the IDMI energy density of a Néel skyrmion [41]. *D* > 0 (*D* < 0) corresponds to the case of the heavy metal under (over) the ferromagnetic material. In general, for the proper skyrmion chirality either for a Néel or a Bloch skyrmions, the DMI energy lowers the skyrmion energy.

#### 2.1.3. Anisotropy Energy Density

The anisotropy energy density gets contributions from the perpendicular uniaxial anisotropy and the demagnetization (magnetostatic) energy densities, respectively. The former term takes the compact form $\varepsilon_{\mathrm{ani}}^{}=-K_{u}m_{z}^{2}$, $K_{u}$ being the perpendicular uniaxial anisotropy constant. In explicit form expressed as a function of *θ* it reads *ε*_ani_ = −*K*_u_ cos^2^*θ*. Instead, the latter term takes the form *ε*_dem_ = −½ *µ*_0_*M*_s_ **m**· **H**_dem_, with **H**_dem_ = (0,0,*H*_dem_) being aligned along +*z* and *µ*_0_ = 4*π* × 10^−7^ H/m being the vacuum permeability. Since skyrmions that are hosted in ultrathin ferromagnetic dots are studied, the ultrathin approximation is applied, according to which the magnetostatic source is given only by the face surface charges of the dot, *H*_dem_ = −*M*_s_ *m_z_*. Indeed, according to this approximation, in the ultrathin limit the contributions resulting from the side surface charges of the dot and from the volume charges can be safely neglected. This leads to εdem=12μ0Ms2cos2θ that has the form of a local term. The total anisotropy energy density can be written as an effective anisotropy by defining an effective anisotropy constant Keff=Ku−12μ0 Ms 2 so that $\;\varepsilon_{\mathrm{ani}}^{\mathrm{eff}}=-K_{\mathrm{eff}}\sin^{2}\theta$. However, for a more realistic description it is convenient to refer the anisotropy energy density to the uniform state (*m* along +*z*), *ε*_ani_ (*θ* =0) = −*K*_u_ obtained for *θ* = 0 expressing it as *ε*_ani_ = *K*_u_ sin^2^*θ*, namely as the difference *ε*_ani_ (*θ*) − *ε*_ani_ (*θ* =0) recovering the definition of ferromagnetic thin films. Hence, *ε*_ani_^eff^ = *K*_u_ − *K*_eff_ cos^2^*θ* and the term proportional to *K*_u_ leads to a constant upshift of the skyrmion energy from negative to positive values due to the high *K*_u_ magnitude for typical ferromagnetic materials without affecting its trend vs. the skyrmion radius.

#### 2.1.4. External Field Energy Density

The Zeeman energy density due to the interaction of the static magnetization with the external bias field **H**_ext_ is *ε*_extfield_ = −*µ*_0_ *M*_s_ **m**·**H**_ext_. In terms of the polar angle *θ* between the magnetization **m** and the *z* axis and the angle $\theta_{\mathrm{Hext}}$ the external bias forms with the *z* axis it is *ε*_extfield_ = −*µ*_0_ *M*_s_ *H*_ext_ cos(*θ*- *θ_H_*_ext_). If **H**_ext_ is aligned along +*z* (–*z*), the Zeeman energy density can be written as *ε*_extfield_ = −*µ*_0_ *M*_s_ *H*_ext_ cos *θ*, with *H*_ext_ > 0 (*H*_ext_ < 0). In both cases, the sign of the Zeeman energy density depends on the sign of cos*θ*, which is in turn related to the **m** orientation with respect the *z* axis.

Instead, if this contribution is expressed as the energy gain with respect to the Zeeman energy density of the perpendicular uniform state (**m** aligned along + *z*, *θ* = 0, with **H**_ext_ aligned along +*z* (*H*_ext_ > 0) or −*z* (*H*_ext_ < 0)) one gets *ε*_extfield_ (*θ*) − *ε*_extfield_ (*θ* = 0) = *µ*_0_ *M*_s_ *H*_ext_ (1 − cos*θ*).

The total energy density is written down using the dimensionless radial coordinate *r* = *ρ/l*_exch_ with *l*_exch_ = √ 2*A*/(*µ*_0_ *M*_s_^2^) being the exchange length. The total energy density for both skyrmion magnetization textures as explicit function of the polar angle *θ* reads:

(2) $$\varepsilon_{\mathrm{tot}}=\tilde{A}\;\left({\left(\frac{d\theta }{dr}\right)}^{2}+\frac{\sin^{2}\theta }{r^{2}}\right)\pm \tilde{D}\;\left(\frac{d\theta }{dr}+\frac{\sin\theta \cos\theta }{r}\right)+K_{u}\sin^{2}\theta +1/2\mu_{0}M_{s}^{2}\cos^{2}\theta -\mu_{0}M_{s}H_{\mathrm{ext}}\cos\theta .$$

with $\tilde{A}=A/l_{{}_{\mathrm{exch}}}^{2}$ and $\tilde{D}=D/l_{\mathrm{exch}}$.

The total energy density in dimensionless units is calculated at equilibrium using the static magnetization distribution *θ*(*r*) = Θ_0_(*r*) at equilibrium of a chiral Néel skyrmion derived in [35]. This distribution is a trial solution of the nonlinear and transcendental differential equation resulting from the minimization of the skyrmion energy functional complemented by the boundary conditions on the distribution itself and its radial derivative. In the limit of dominating exchange isotropic interaction (quality factor *Q* = 1 with *Q* = 2*K*_u_/(*μ*_0_*M*_s_^2^) and DMI parameter *D* = 0), this solution recovers the well-known Belavin–Polyakov soliton solution [42,44]. Θ_0_(*r*) describes the radial dependence of the static magnetization of a chiral Néel skyrmion having static magnetization along –*z* (corresponding to skyrmion number *S* = −1) hosted in a circular dot. It takes the form:

(3a) $$\Theta_{0}(r)=\;2\arctan\left(B\;\frac{e^{-\xi r}}{r}\right)$$

where $\;B=r_{\mathrm{sky}}e^{\xi \;r_{\mathrm{sky}}}$, with *r*_sky_ =*R*_sky_/*l*_exch_ the dimensionless skyrmion radius and *R*_sky_ the skyrmion radius, and $\xi =\sqrt{Q-1}$. According to Equation (3a) it is $\Theta_{0}(r=0)=\pi$(*m*_z_ = −1, static magnetization along −*z* in the core center) and $\Theta_{0}(r\to \infty )=0$(*m*_z_ = +1, static magnetization along +*z* at the skyrmion border).

Analogously, the corresponding magnetization distribution at equilibrium for static magnetization along +*z* with skyrmion number *S* = +1 reads:

(3b) $$\Theta_{0}(r)=\pi -\;2\arctan\left(B\;\frac{e^{\;-\xi r}}{r}\right).$$

According to Equation (3b) it is $\Theta_{0}(r=0)=0$ (*m*_z_ = +1, static magnetization along +*z* in the core center) and $\Theta_{0}(r\to \infty )=\pi$ (*m*_z_ = −1, static magnetization along -*z* at the skyrmion border). Note that the trial magnetization distribution expressed by Equation (3) is the solution of the variational nonlinear differential equation given in [35]. This trial solution is complemented by the boundary conditions Θ_0_(*r* = 0) =0,*π*, Θ_0_(*r* = *r*_sky_) =*π/2* and $\frac{\partial \Theta_{0}}{\partial r}(r=r_{d})=\frac{\left|D\right|l_{\mathrm{exch}}}{2A}$ with *r*_d_ = *R*_d_/*l*_exch_ the dimensionless dot radius (*R*_d_ is the dot radius) and is valid also for a Bloch skyrmion because the variational differential equation takes the same form (the bulk DMI energy density has indeed the same expression as the IDMI energy density) and the boundary conditions are the same. In principle, these boundary conditions do not take into account the canting of the magnetization at the borders that would become important when the skyrmion radius is comparable to the dot radius. However, in the analytical and numerical calculations presented and discussed in Section 3, this issue does not arise. Indeed, the equilibrium skyrmion radius calculated in the metastability region for vanishing external bias field and in the presence of **H**_ext_ is at most less than ¼ of the dot radius for all the cases examined including the comparison of the numerical calculations with the experimental data.

From Equations (3a) and (3b) one gets:

(3c) $$\frac{d\Theta_{0}(r)}{dr}=\mp \frac{2B(1+r\xi )e^{-\xi r}}{r^{2}+B^{2}e^{-2\xi r}}$$

where the minus (plus) sign is referred to the skyrmion magnetization texture with the static magnetization along -*z* (+*z*) in the core center corresponding to skyrmion number *S* = −1 (*S* = +1). Substituting Equation (3) in Equation (2) yields:

(4) $$\begin{array}{l} \varepsilon_{\mathrm{tot}}=\tilde{A}\left[{\left(\mp \frac{2B(1+r\xi )e^{-\xi r}}{r^{2}+B^{2}e^{-2\xi r}}\right)}^{2}+\frac{\sin^{2}\left(2\arctan\left(B\;\frac{e^{\;-\xi r}}{r}\right)\right)}{r^{2}}\right]\pm \tilde{D}\left[\pm \frac{\sin\left(2\arctan\left(B\;\frac{e^{-\xi r}}{r}\right)\right)\cos\left(2\arctan\left(B\;\frac{e^{\;-\xi r}}{r}\right)\right)}{r}\mp \frac{2B(1+r\xi )e^{-\xi r}}{r^{2}+B^{2}e^{-2\xi r}}\right]\\ +K_{u}-K_{\mathrm{eff}}\cos^{2}\left(2\arctan\left(B\;\frac{e^{-\xi r}}{r}\right)\right)\mp \mu_{0}M_{s}H_{\mathrm{ext}\;}\cos\left(2\arctan\left(B\;\frac{e^{-\xi r}}{r}\right)\right). \end{array}$$

Here, the upper (lower) signs in the first term of the exchange energy density and in the two terms of the DMI energy density refer to the magnetization texture with the static magnetization along −*z* (+*z*) in the core center (*r* = 0) corresponding to *S* = −1 (*S* = +1). Instead, the upper (lower) sign in the Zeeman energy density refers to the static magnetization *m_z_* along −*z* (+*z*) in the core center (*r* = 0) and along +*z* (−*z*) at the skyrmion border being $\cos\left(\pi -2\arctan\left(B\;\frac{e^{-\xi r}}{r}\right)\right)=-\cos\left(2\arctan\left(B\;\frac{e^{\;-\xi r}}{r}\right)\right)$ and the external magnetic field **H**_ext_ applied either along +*z* (*H*_ext_ > 0) or along –*z* (*H*_ext_ < 0). In other words, the “−“ (“+”) sign in front of the Zeeman energy density contribution refers to the cases where the static magnetization *m_z_* (*r* = 0) is anti-parallel (parallel) to **H**_ext_. By using some trigonometric expansions (see the Appendix A, Equations (A1)–(A4)), one gets:

(5) $$\begin{array}{l} \varepsilon_{\mathrm{tot}}\left(r,r_{\mathrm{sky}}\right)=4\tilde{A}\;B^{2}\left[\frac{\;\left(2+2r\xi +r^{2}\xi^{2}\right)e^{-2\xi r}}{{\left(r^{2}+B^{2}e^{-2\xi r}\right)}^{2}}\right]\pm 2\tilde{D}\;B\left[\pm \frac{\left(r^{2}-B^{2}\;e^{\;-2\xi r}\right)e^{-\xi r}}{{\left(r^{2}+B^{2}e^{-2\xi r}\right)}^{2}}\mp \frac{(1+r\xi )e^{-\xi r}}{r^{2}+B^{2}e^{-2\xi r}}\right]\\ +K_{u}-K_{\mathrm{eff}}{\left(\frac{r^{2}-B^{2}e^{-2\xi r}}{r^{2}+B^{2}e^{-2\xi r}}\right)}^{2}\mp \mu_{0}M_{s}H_{\mathrm{ext}}\frac{r^{2}-B^{2}e^{-2\xi r}}{r^{2}+B^{2}e^{-2\xi r}}. \end{array}$$

Equation (5) is Equation (A5) of the Appendix A. The expression of the total energy density given by Equation (5) is valid for any Néel and Bloch magnetic skyrmion either for chirality *χ* = +1 (+ sign in front of the DMI term) or *χ* = −1 (− sign in front of the DMI term). Here, it is *H*_ext_ > 0 (*H*_ext_ < 0) if the external magnetic field is along +*z* (−*z*). For *D* > 0, it is always *m_z_* along −*z* in the core center, *r* = 0 (*S* = −1), and *χ* = +1 (radially outward Néel skyrmion and counter-clockwise Bloch skyrmion).

### 2.2. Skyrmion Energy

In this subsection the calculation of the skyrmion total energy *E* is outlined. The energy density is integrated over the dot volume $E\left(r_{\mathrm{sky}}\right)=\int \varepsilon_{\mathrm{tot}}\left(r,r_{\mathrm{sky}}\right)dV$ that, in explicit form, is $E\left(r_{\mathrm{sky}}\right)=\int \varepsilon_{\mathrm{tot}}\left(r,r_{\mathrm{sky}}\right)dV={\left(l_{\mathrm{exch}}\right)}^{2}\int_{-t/2}^{t/2}dz\int_{0}^{2\pi }d\phi \int_{0}^{r_{d}\;}\varepsilon_{\mathrm{tot}}\left(r,r_{\mathrm{sky}}\right)r\;dr$ so that:

(6) $$E\left(r_{\mathrm{sky}}\right)=2\pi t\;{\left(l_{\mathrm{exch}}\right)}^{2}\int_{0}^{\;r_{d}}\varepsilon_{\mathrm{tot}}\left(r,r_{\mathrm{sky}}\right)r\;dr$$

where *t* is the dot thickness.

The total energy expressed in Equation (6) is computed starting from the energy density of Equation (5). The total energy consists of the following energy contributions: (1) ferromagnetic exchange; (2) DMI (either IDMI or bulk DMI); (3) effective anisotropy; and (4) contribution dependent on the external bias field. The following dimensionless integrals are evaluated analytically: (1) three $I_{i\text{-}\mathrm{exch}}$ for the ferromagnetic exchange; (2) four $I_{i\text{-}\mathrm{DMI}}$ for the IDMI; (3) $I_{\mathrm{ani}}$ for the effective perpendicular anisotropy; and (4) $I_{\mathrm{extfield}}$ for the contribution dependent on the external bias field. All integrals have a dependence on the skyrmion radius *r*_sky_. In compact form:

(7) $$E\left(r_{\mathrm{sky}}\right)=2\pi t\;{\left(l_{\mathrm{exch}}\right)}^{2}\left(4\tilde{A}\;\sum_{i=1}^{3}\;I_{i\text{-exch}}\pm 2\tilde{D}\;\sum_{i=1}^{4}I_{i\text{-DMI}}\;+K_{u}V-K_{\mathrm{eff}}I_{\mathrm{ani}}-\mu_{0}M_{s}H_{\mathrm{ext}}I_{\mathrm{extfield}}\right)$$

with *E* = *E*(*r*_sky_). The explicit expressions of the integrals appearing in Equation (7) are in the Appendix A (Equations (A6)–(A14)). For every contribution of the total skyrmion energy, the radial integration is carried out based on two substitutions. The first substitution is a change of variable: (1) *ξ r = s*. This leads to *e^ξ r^* = *e^s^* and to *r* = 1/*ξ s*. The second substitution is a real substitution based on the change of variable given in 1): (2) *s e^s^* = *x* so that *x* = *ξ r e^ξr^*. The transcendental equation *s e^s^* = *x* admits the solution *s* = *W*(*x*), allowing to write *W*(*x*) *e^W(x)^* = *x*, where *W*(*x*) is the Lambert function defined in the real domain as a function of the real variable *x* and *ds* = *W*(*x*)/(*x* (1 + *W*(*x*))) *dx*, with *W*(0) = 0. This allows writing every integral in terms of the variable *x*, with 0 ≤ *x* ≤ *C* and $C=\xi r_{d}e^{\xi }{}^{r_{d}}$ a quantity depending on the dot radius. Hence, in these calculations the domain of the Lambert function is restricted to the interval 0 ≤ *x* ≤ *C*, where *W(x*) ≥ 0.

Hence, all the integrand functions can be written as the product of a rational fractional function *f* = *f* (*B*,*x*) and a fractional function *g*(*W*(*x*)) dependent on *W*(*x*). While the former function has an explicit dependence on the skyrmion radius *r*_sky_ via *B* and on the radial coordinate *r,* the latter function has an explicit dependence only on the radial coordinate *r* being *x* = *ξ r e^ξ r^*. The generic integral *I* for every skyrmion energy contribution is expressed in the form:

(8a) $$I=K\left(B,\xi \right)\int_{0}^{C}f\left(B,x\right)g\left(W\left(x\right)\right)dx$$

where *K* = *K*(*B*,*ξ*) is a coefficient that is dependent on *B* and *ξ* and assuming different forms depending on the considered integral and *g*(*W*(*x*)) is either *g*(*W*(*x*)) = *W*(*x*)/(1 + *W*(*x*)) or *g*(*W*(*x*)) = (*W*(*x*))^2^/(1 + *W*(*x*)) depending on the considered integral.

**Figure 1:**
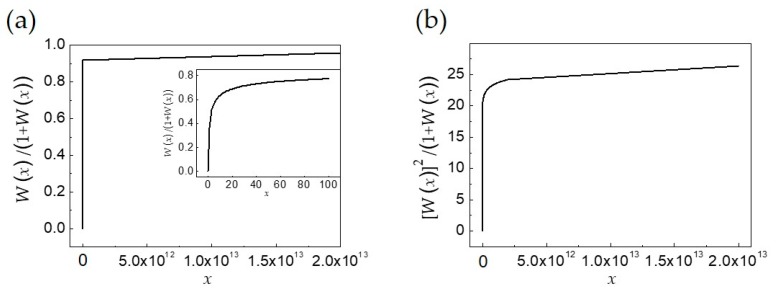
(**a**) Plot of *W*(*x*)/(1 + *W*(*x*)) function for the whole interval of integration 0 ≤ *x* ≤ *C*, with *C* = 2.0 × 10^13^. Inset: Plot of *W*(*x*)/(1 + *W*(*x*)) function in the interval 0 ≤ *x* ≤ 100. (**b**) As in (**a**) but for [*W*(*x*)]^2^/(1 + *W*(*x*)).

The function *g*(*W*(*x*)) appearing in the integral of Equation (8a) is plotted in Figure 1 for 0 ≤ *x ≤ C* for the two different functions appearing in the skyrmion energy integrals (see the next subsections for the details). In Figure 1a the function *W*(*x*)/(1 + *W*(*x*)) is shown, while in Figure 1b it is the function (*W*(*x*))^2^/(1 + *W*(*x*)). In the skyrmion energy integrals (see Section 2.2.1 for the details) it appears either *W*(*x*)/(1 + *W*(*x*)) or (*W*(*x*))^2^/(1 + *W*(*x*)), depending on the integral. For the special case shown corresponding to the parameters at *T* = 0 K (see Section 3 for their numerical values) *C* = 2 × 10^13^. Both functions vanish for *x* = 0, are monotonically increasing functions but, while *W*(*x*)/(1 + *W*(*x*)) tends asymptotically to 1 for increasing *x*, (*W*(*x*))^2^/(1 + *W*(*x*)) diverges for *x* → ∞, exhibiting a trend similar to that of *W*(*x*). The smooth behavior of *W*(*x*)/(1 + *W*(*x*)) for small *x* that is masked by the large *x* interval is shown in more detail in the inset to Figure 1a for a reduced interval of *x* (0 ≤ *x ≤* 100).

Because of the rescaled parameters used at higher temperatures, a reduction of the value of *C* occurs without altering the qualitative behavior of the two functions.

The generic integrand function *h*(*x*) of Equation (8a) does not have a primitive function for every considered energy contribution, therefore the integral *I* cannot be solved analytically. However, from a numerical check studying the behavior of the integrand functions, it has been found that, for every energy contribution, the integral *I* is well approximated if calculated in the form:

(8b) $$I≃K\left(B,\xi \right)g\left(W\left(B\;\xi \right)\right)\int_{0}^{C}f\left(B,x\right)dx$$

namely, moving out of the integral the function *g*(*W*(*x*)) depending on the radial coordinate only, and attributing to it a trend depending on the skyrmion radius via the dependence on *B* (with *B* = *B* (*r*_sky_)) and writing *g*(*W*(*Bξ*)) in place of *g*(*W*(*x*)). In this way, the radial dependence contained in *g*(*W*(*x*)) has been interchanged with the skyrmion radius dependence. This has been accomplished, first by numerically calculating the integral of Equation (8b) whose integrand *f*(*B*,*x*) has a primitive function for every energy contribution of Equation (7) (see the next subsections), and then by numerically computing the ratio between the numerical integral of Equation (8a) and the integral of Equation (8b), both depending on *r*_sky_ for the values of the skyrmion radius in the interval 0 ≤ *r*_sky_ ≤ *r*_d_. The calculation was done for every energy contribution of Equation (7). The above-mentioned ratio turned out to be approximately equal to the function *g*(*W*(*Bξ*)) for all skyrmion radii ranging in the interval 0 ≤ *r*_sky_ ≤ *r*_d_. In particular, *g*(*W*(*Bξ*)) = *W*(*Bξ*)/(1 + *W*(*Bξ*)), *g*(*W*(*Bξ*)) = [*W*(*Bξ*)]^2^/(1 + *W*(*Bξ*)), or *g*(*W*(*Bξ*)) =1/(2*ξ*^3^) [*W*(*Bξ*)]^2^/(1 + *W*(*Bξ*)), with 0 ≤ *B* ≤ 1/*ξ C* depending on the integral studied (see the following subsection for the details).

#### 2.2.1. Calculation of Ferromagnetic Exchange Energy

In this subsection, the ferromagnetic exchange energy is computed. Taking into account substitution (1), multiplying numerator and denominator by *e*^4*s*^, and substitution (2) $I_{1\text{-exch}}$ is rewritten (Equation (A6)) as:

(9a) $$I_{1\text{-exch}}=2{\left(B\xi \right)}^{2}\int_{0}^{C}\frac{x\;}{{\left(x^{2}+{\left(B\xi \right)}^{2}\right)}^{\;2}}\frac{1}{1+W\left(x\right)}dx.$$

Taking into account substitution (1), multiplying numerator and denominator by *e*^4*s*^, and substitution (2), $I_{2\text{-exch}}$ (Equation (A7)) is rewritten as:

(9b) $$I_{2\text{-exch}}=2{\left(B\xi \right)}^{2}\int_{0}^{C}\frac{x\;}{\;{\left(x^{2}+{\left(B\xi \right)}^{2}\right)}^{{}^{2}}}\frac{W\left(x\right)}{1+W\left(x\right)}dx.$$

Now, by considering the sum *I*_1-2exch_ of the two integrals of Equations (9a) and (9b) that does not depend on *W*(*x*):

(9c) $$I_{1\text{-}2\text{-exch}}=2{\left(B\xi \right)}^{2}\int_{0}^{C}\frac{x\;}{\;{\left(x^{2}+{\left(B\xi \right)}^{2}\right)}^{\;2}}dx.$$

Performing the integral of Equation (9c) one gets:

(9d) $$I_{1\text{-}2\text{-exch}}=\frac{1}{1+{\left(P\xi \right)}^{2}\;}$$

with *P* = *B*/*C* and, in explicit form, $P=\frac{r_{\mathrm{sky}}}{\xi \;r_{d}}e^{\xi \left(r_{\mathrm{sky}}-r_{d}\right)}$. Hence, *P* does depend on *r*_sky_ that is a variable quantity.

Finally, taking into account substitution (1), multiplying numerator and denominator by *e*^4*s*^, and substitution (2), *I*_3-exch_ (Equation (A8)) becomes:

(10a) $$I_{3\text{-}\mathrm{exch}}={\left(B\xi \right)}^{2}\int_{0}^{C}\frac{x\;}{\;{\left(x^{2}+{\left(B\xi \right)}^{2}\right)}^{\;2}}\frac{{\left[W\left(x\right)\right]}^{\;2}}{1+W\left(x\right)}dx.$$

Owing to the approximation of Equation (8b) the integral can be rewritten in the form:

(10b) $$I_{3\text{-}\mathrm{exch}}≃b{\left(B\xi \right)}^{2}\int_{0}^{C}\frac{x\;}{\;{\left(x^{2}+{\left(B\xi \right)}^{2}\right)}^{\;2}}dx$$

with $b=\frac{{\left[W\left(C\;P\;\xi \right)\right]}^{\;2}}{1+W\left(C\;P\xi \right)}$. Solving the integral yields:

(10c) $$I_{3\text{-exch}}≃\frac{1}{2}\frac{{\left[W\left(C\;P\;\xi \right)\right]}^{\;2}}{1+W\left(C\;P\xi \right)}\frac{1}{1+{\left(P\xi \right)}^{2}\;}.$$

The corresponding exchange energy $E_{\mathrm{exch}}=8\pi t\;A\;I_{\mathrm{exch}}$ with $I_{\mathrm{exch}}=\sum_{i=1}^{3}\;I_{i\text{-}\mathrm{exch}}$ reads:

(11a) $$E_{\mathrm{exch}}≃8\pi tA\left(1+\frac{1}{2}\frac{{\left[W\left(C\;P\;\xi \right)\right]}^{\;2}}{1+W\left(C\;P\xi \right)}\right)\frac{1}{1+{\left(P\xi \right)}^{2}\;}.$$

As *r*_sky_→0, *B* and *P*→0 so that:

(11b) $$E_{\mathrm{exch}}\left(r_{\mathrm{sky}}\to 0\right)=8\pi \;t\;A.$$

Hence, the limit 8*π t A* is recovered representing the absolute minimum of the exchange energy of a continuous spin structure with integer topological charge [42,44].

#### 2.2.2. Calculation of DMI Energy

This subsection is devoted to the calculation of DMI energy. As shown above this calculation is valid for both IDMI and bulk DMI energy. Taking into account substitution (1), multiplying the numerator and denominator by *e*^4*s*^, and substitution (2), *I*_1-DMI_ (Equation (A9)) becomes:

(12a) $$I_{1\text{-DMI}}=\pm B^{}\int_{0}^{C}\frac{x^{2}\;}{\;{\left[x^{2}+{\left(B\xi \right)}^{2}\right]}^{\;2}}\frac{W\left(x\right)}{1+W\left(x\right)}dx.$$

Because of the approximation of Equation (8b) *I*_1-DMI_ can be rewritten as:

(12b) $$I_{1\text{-DMI}}≃\pm c\;B^{}\int_{0}^{C}\frac{x^{2}\;}{\;{\left(x^{2}+{\left(B\xi \right)}^{2}\right)}^{\;2}}dx$$

with $c=\frac{W\left(C\;P\;\xi \right)}{1+W\left(C\;P\xi \right)}$. The computation of the integral leads to:

(12c) $$I_{1\text{-DMI}}≃\pm \frac{1}{2\xi }\frac{W\left(C\;P\;\xi \right)}{1+W\left(C\;P\xi \right)}\;\left(\mathrm{arccotg}\left(P\xi \right)-\frac{P\xi }{1+{\left(P\xi \right)}^{2}}\right).$$

Taking into account substitution (1), multiplying numerator and denominator by *e*^4*s*^, and substitution (2), *I*_2-DMI_ (Equation (A10)) becomes:

(13a) $$I_{2\text{-DMI}}=\mp B^{3}\xi^{2}\int_{0}^{C}\frac{1\;}{\;{\left(x^{2}+{\left(B\xi \right)}^{2}\right)}^{2}}\frac{W\left(x\right)}{1+W\left(x\right)}dx.$$

According to the approximation of Equation (8b) the integral can be rewritten in the form:

(13b) $$I_{2\text{-DMI}}≃\mp B^{3}\xi^{2}c\int_{0}^{C}\frac{1\;}{\;{\left(x^{2}+{\left(B\xi \right)}^{2}\right)}^{2}}dx.$$

Carrying out the integration yields:

(13c) $$I_{2\text{-DMI}}≃\mp \frac{1}{2\xi }\frac{W\left(C\;P\;\xi \right)}{1+W\left(C\;P\xi \right)}\;\left(\mathrm{arccotg}\left(P\xi \right)+\frac{P\xi }{1+{\left(P\xi \right)}^{2}}\right).$$

Taking into account substitution (1), multiplying numerator and denominator by *e*^2*s*^, and substitution (2), *I*_3-DMI_ (Equation (A11)) reads:

(14a) $$I_{3\text{-DMI}}=\mp B\;\int_{0}^{C}\frac{1\;}{\;x^{2}+{\left(B\xi \right)}^{2}}\frac{W\left(x\right)}{1+W\left(x\right)}dx.$$

The integral is rewritten in the form:

(14b) $$I_{3\text{-DMI}}≃\mp c\;B\int_{0}^{C}\frac{1\;}{\;x^{2}+{\left(B\xi \right)}^{2}}dx.$$

Solving the integral yields:

(14c) $$I_{3\text{-DMI}}≃\mp \frac{1}{\xi }\frac{W\left(C\;P\;\xi \right)}{1+W\left(C\;P\xi \right)}\;\mathrm{arccotg}\left(P\xi \right)\;.$$

Taking into account substitution (1), multiplying numerator and denominator by *e*^2*s*^, and substitution (2), $I_{4\text{-}I\mathrm{DMI}}$ (Equation (A12)) is rewritten as:

(15a) $$I_{4\text{-DMI}}=\mp B\int_{0}^{C}\frac{\;1}{x^{2}+{\left(B\xi \right)}^{2}}\frac{{\left[W\left(x\right)\right]}^{\;2}}{1+W\left(x\right)}dx$$

where the −(+) in front refers to chirality *χ* = +1 (*χ* = −1). The integral can be approximated in the form taking into account the approximation of Equation (8b):

(15b) $$I_{4\text{-DMI}}≃\mp bB\int_{0}^{C}\frac{\;1\;}{x^{2}+{\left(B\xi \right)}^{2}}dx.$$

The calculation of the integral yields:

(15c) $$I_{4\text{-DMI}}≃\mp \frac{1}{\xi }\;\frac{{\left[W\left(C\;P\;\xi \right)\right]}^{2}}{1+W\left(C\;P\xi \right)}\mathrm{arccotg}\left(P\xi \right)\;.$$

The total DMI energy is expressed as *E*_DMI_ = ±4*π t D l*_exch_ *I*_DMI_ with $\;I_{\mathrm{DMI}}=\sum_{i=1}^{4}I_{i\text{-}\mathrm{DMI}}$ and reads:

(16) $$E_{\mathrm{DMI}}≃\pm \left(4\pi t\;D\;l_{\mathrm{exch}}\right)\left[\mp \frac{1}{\xi }\;\left(\left(\frac{{\left[W\left(C\;P\;\xi \right)\right]}^{2}+W\left(C\;P\;\xi \right)}{1+W\left(C\;P\xi \right)}\right)\;\mathrm{arccotg}\left(P\xi \right)+\frac{W\left(C\;P\;\xi \right)}{1+W\left(C\;P\xi \right)}\left(\frac{P\xi }{1+{\left(P\xi \right)}^{2}}\right)\right)\;\right]$$

with *b* = *b* (*P*) and *c* = *c* (*P*).

#### 2.2.3. Calculation of the Effective Anisotropy Energy

Taking into account substitution (1), multiplying numerator and denominator by *e*^2*s*^, and substitution (2), $I_{\mathrm{ani}}^{\mathrm{eff}}$ (Equation (A13)) turns out to be:

(17a) $$I_{\mathrm{ani}}^{\mathrm{eff}}=\frac{1}{\xi^{2}}\int_{0}^{C}\frac{\;{\left(x^{2}-{\left(B\xi \right)}^{2}\right)}^{\;2}}{x{\left(x^{2}+{\left(B\xi \right)}^{2}\right)}^{\;2}}\frac{{\left[W\left(x\right)\right]}^{\;2}}{1+W\left(x\right)}dx.$$

Adding and subtracting 4(*Bξ*)^2^ *x*^2^ at the numerator of the integrand function yields:

(17b) $$I_{\mathrm{ani}}^{\mathrm{eff}}=\frac{1}{\xi^{2}}\left[\int_{0}^{C}\frac{\;1}{x}\frac{{\left[W\left(x\right)\right]}^{\;2}}{1+W\left(x\right)}dx-4\int_{0}^{C}\frac{\;{\left(B\xi \right)}^{2}x}{{\left(x^{2}+{\left(B\xi \right)}^{2}\right)}^{\;2}}\frac{{\left[W\left(x\right)\right]}^{\;2}}{1+W\left(x\right)}dx\right].$$

In particular:

(17c) $$I_{\mathrm{ani}1}^{\mathrm{eff}}=\frac{1}{\xi^{2}}\int_{0}^{C}\frac{\;1}{x}\frac{{\left[W\left(x\right)\right]}^{\;2}}{1+W\left(x\right)}dx$$

and:

(17d) $$I_{\mathrm{ani}2}^{\mathrm{eff}}=-\frac{4}{\xi^{2}}\int_{0}^{C}\frac{\;{\left(B\xi \right)}^{2}x}{{\left(x^{2}+{\left(B\xi \right)}^{2}\right)}^{\;2}}\frac{{\left[W\left(x\right)\right]}^{\;2}}{1+W\left(x\right)}dx.$$

$I_{\mathrm{ani}1}^{\mathrm{eff}}$ is solved exactly yielding:

(17e) $$I_{\mathrm{ani}1}^{\mathrm{eff}}=\frac{1}{2\xi^{2}}{\left[W\left(C\right)\right]}^{\;2}.$$

Because of the approximation of Equation (8b), $I_{\mathrm{ani}2}^{\mathrm{eff}}$ is rewritten as:

(17f) $$I_{\mathrm{ani}2}^{\mathrm{eff}}≃-\frac{4}{\xi^{2}}b\int_{0}^{C}\frac{\;{\left(B\xi \right)}^{2}x}{{\left(x^{2}+{\left(B\xi \right)}^{2}\right)}^{\;2}}dx.$$

Solving the integral one gets:

(17g) $$I_{\mathrm{ani}2}^{\mathrm{eff}}≃-\frac{1}{\xi^{2}}\frac{{\left[W\left(C\;P\xi \right)\right]}^{2}}{1+W\left(C\;P\xi \right)}\left(\frac{2}{1+{\left(P\xi \right)}^{2}}\right)$$

with $I_{\mathrm{ani}}^{\mathrm{eff}}=I_{\mathrm{ani}1}^{\mathrm{eff}}+I_{\mathrm{ani}2}^{\mathrm{eff}}>0$. Substituting Equations (17e) and (17g), the effective anisotropy energy *E*_ani_ = −2*π* (*l*_exch_)^2^*t K*_eff_$I_{\mathrm{ani}}^{\mathrm{eff}}$ reads:

(18) $$E_{{}_{\mathrm{ani}}}^{\mathrm{eff}}≃-\pi K_{\mathrm{eff}}t{\left(l_{\mathrm{exch}}\right)}^{2}\frac{1}{\xi^{2}}\left[{\left[W\left(C\right)\right]}^{2}-\frac{{\left[W\left(CP\xi \right)\right]}^{2}}{\left(1+W\left(CP\xi \right)\right)}\frac{4}{\left(1+{\left(P\xi \right)}^{2}\right)}\right].$$

This term must be added the quantity *K*_u_ *V* that leads to a rigid shift of the skyrmion internal energy.

#### 2.2.4. Calculation of the External Field Energy

Taking into account substitution (1), multiplying numerator and denominator by *e*^2*s*^, and substitution (2), *I*_extfield_ (Equation (A14)) takes the form:

(19a) $$I_{\mathrm{extfield}}=\frac{1}{\xi^{2}}\int_{0}^{C}\frac{1}{x}\left(\frac{\;x^{2}-{\left(B\xi \right)}^{2}}{x^{2}+{\left(B\xi \right)}^{2}}\right)\frac{{\left[W\left(x\right)\right]}^{\;2}}{1+W\left(x\right)}dx.$$

Adding and subtracting 2(*Bξ*)^2^ at the numerator of the integrand function yields:

(19b) $$I_{\mathrm{extfield}}=\frac{1}{\xi^{2}}\left[\int_{0}^{C}\frac{1}{x}\frac{{\left[W\left(x\right)\right]}^{\;2}}{1+W\left(x\right)}dx-\int_{0}^{C}\frac{2}{x}\frac{\;{\left(B\xi \right)}^{2}}{x^{2}+{\left(B\xi \right)}^{2}}\frac{{\left[W\left(x\right)\right]}^{\;2}}{1+W\left(x\right)}dx\right].$$

In particular:

(19c) $$I_{\mathrm{extfield}1}=\frac{1}{\xi^{2}}\int_{0}^{C}\frac{1}{x}\frac{{\left[W\left(x\right)\right]}^{\;2}}{1+W\left(x\right)}dx$$

and:

(19d) $$I_{\mathrm{extfield}2}=-\frac{2}{\xi^{2}}\int_{0}^{C}\frac{1}{x}\frac{\;{\left(B\xi \right)}^{2}}{x^{2}+{\left(B\xi \right)}^{2}}\frac{{\left[W\left(x\right)\right]}^{\;2}}{1+W\left(x\right)}dx.$$

*I*_extfield1_ is solved exactly yielding:

(19e) $$I_{\mathrm{extfield}1}=\frac{1}{2\xi^{2}}{\left[W\left(C\right)\right]}^{\;2}.$$

Owing to the approximation of Equation (8b), singling out the divergence of the integrand function in *x* = 0 and checking numerically the integral of Equation (19d), *I*_extfield2_ is rewritten as proportional to the coefficient *b* weighted by 1/(2*ξ*^3^):

(19f) $$I_{\mathrm{extfield}2}≃\;-\;\frac{1}{2\xi^{3}}b\frac{2}{\xi^{2}}\int_{\varepsilon }^{C}\frac{1}{x}\frac{\;{\left(B\xi \right)}^{2}}{x^{2}+{\left(B\xi \right)}^{2}}dx.$$

with *ε→*0 and *x*ϵ (*ε*,*C*). Solving the integral one gets:

(19g) $$I_{\mathrm{extfield}2}≃-\frac{1}{2\xi^{5}}\frac{{\left[W\left(CP\xi \right)\right]}^{\;2}}{1+W\left(CP\xi \right)}\left(\ln\left(\frac{{\left(P\xi \right)}^{2}}{1+{\left(P\xi \right)}^{2}}\right)+2\;\ln\left(\frac{C}{\varepsilon }\right)\right).$$

The Zeeman energy due to the external bias field reads:

(20) $$E_{\mathrm{extfield}}≃\mp \pi t\;{\left(l_{\mathrm{exch}}\right)}^{2}\mu_{0}M_{s}H_{\mathrm{ext}}\frac{1}{\xi^{2}}\left[{\left[W\left(C\right)\right]}^{\;2}-\frac{1}{\xi^{3}}\frac{{\left[W\left(CP\xi \right)\right]}^{\;2}}{1+W\left(CP\xi \right)}\left(\ln\left(\frac{{\left(P\;\xi \right)}^{2}}{1+{\left(P\;\xi \right)}^{2}}\right)+2\ln\left(\frac{C}{\varepsilon }\right)\right)\right].$$

Attention must be paid to the computation of *E*_extfield_ because of the last term strictly depending on the value of *ε* with *ε→*0. In the numerical calculations, from the comparison with the exact calculation it is found that *ε* = 0.001. The total energy *E* = *E*_exch_ + *E*_IDMI_ + *E*_ani_ + *E*_extfield_ of the magnetic chiral skyrmion (either Néel or Bloch magnetization texture) in the presence of a perpendicular external magnetic field is the sum of the energy contributions appearing in Equations (11a), (16), (18), and (20). It has been shown that, according to the most accurate magnetization distribution for chiral skyrmions hosted in ultrathin cylindrical dots available, the total energy can be expressed as a combination of transcendental functions.

As magnetic parameters *M*_s_, *A*, *D*, and *K*_u_ vary with *T* according to the following scaling laws *A*(*m*) = *A* (*T* = 0 K) *m* (*T*)^3/2^, *D* (*T*) = *D*(*T* = 0 K) *m*(*T*)^3/2^, and *K*_u_ (*T*) = *K*_u_ (*T* = 0 K) *m*(*T*)^3^*^.^*^6^ [35] also *ξ* = *ξ* (*T*), the exchange *l*_exch_ = *l*_exch_ (*T*), the upper integral limit *C* = *C*(*T*). Moreover, the total skyrmion energy is *E* = *E* (*T*), an internal energy depending on temperature. Note that the thermal effects incorporated in the temperature-dependent magnetic parameters affect also the skyrmion modes. Indeed, they activate not only the skyrmion breathing mode, the only mode that preserves the skyrmion symmetry, but also other skyrmion modes such as, e.g., the skyrmion translational mode that, in principle, breaks the skyrmion symmetry.

The skyrmion radius can be obtained by minimizing the internal energy *E*, *∂E*/*∂r*_sky_ = 0, and *∂*^2^*E*/*∂r*_sky_^2^ > 0. For the sake of convenience, all terms of the skyrmion energy are divided by 8 *π t A* setting (*D*/2*A*) *l*_exch_ = *α*, (*K*_eff_/8 *A*) (*l*_exch_)^2^ = *β*, and (*μ*_0_*M*_s_*H*_ext_/8*A*) (*l*_exch_)^2^ = *γ*, with *α*, *β* > 0 and *γ* > 0 (*γ* < 0) if *H*_ext_ > 0, along +*z* (if *H*_ext_ < 0, along −*z*). The energy minimization condition is rewritten as ∂*g*(*P*)/∂*r*_sky_ = 0, with the dimensionless quantity *g*(*P*) = *E*/(8 *π t A*) having the meaning of a reduced energy given by:

(21a) $$\begin{array}{l} g\left(P\right)=\left(1+\frac{1}{2}b\right)\frac{1}{1+{\left(P\xi \right)}^{2}\;}\pm \frac{\alpha }{\xi }\left(\mp \left(b+c\right)\;\mathrm{arccotg}\left(P\xi \right)\mp c\frac{P\xi }{1+{\left(P\xi \right)}^{2}}\right)\\ -\frac{\beta }{\xi^{2}}\left({\left[W\left(C\right)\right]}^{2}-b\frac{4}{1+{\left(P\xi \right)}^{2}}\right)\mp \frac{\gamma }{\xi^{2}}\left({\left[W\left(C\right)\right]}^{\;2}-\frac{1}{\xi^{3}}b\left(\ln\left(\frac{{\left(P\;\xi \right)}^{2}}{1+{\left(P\;\xi \right)}^{2}}\right)-\;\ln{\left(\frac{\varepsilon }{C}\right)}^{2}\right)\right) \end{array}$$

where the + (−) sign in front of the coefficient *α/ξ*^2^ multiplies the − (+) signs inside the round brackets, *b* = *b* (*CPξ*), *c* = *c* (*CPξ*), and the − (+) sign in front of the coefficient *γ/ξ*^2^ refers to *m_z_* = −1 (*m_z_* = +1) in the core center (*r* = 0) and *m*_z_ = +1 (*m_z_* = −1) at the skyrmion border (corresponding to *S* = −1 (*S* = +1)).

Since it has been found from the numerical calculations that the equilibrium skyrmion radius, *r*_sky_ (*E* = *E*_min_) << *r*_d_ for the range of temperatures investigated with *E*_min_ the skyrmion energy minimum, *b* is expanded to the second order, viz. *b* ≈ (*CPξ*)^2^ and *c* to the first order, viz. *c* ≈ *CPξ* getting:

(21b) $$\begin{array}{l} g\left(P\right)≃\left(1+\frac{1}{2}{\left(CP\xi \right)}^{2}\right)\frac{1}{1+{\left(P\xi \right)}^{2}\;}\pm \frac{\alpha }{\xi }\left(\mp \left(C^{2}{\left(P\xi \right)}^{2}+CP\xi \right)\;\mathrm{arccotg}\left(P\xi \right)\mp \frac{C{\left(P\xi \right)}^{2}}{1+{\left(P\xi \right)}^{2}}\right)\\ -\frac{\beta }{\xi^{2}}\left({\left[W\left(C\right)\right]}^{2}-C^{2}{\left(P\xi \right)}^{2}\frac{4}{1+{\left(P\xi \right)}^{2}}\right)\mp \frac{\gamma }{\xi^{2}}\left({\left[W\left(C\right)\right]}^{\;2}-\frac{1}{\xi^{3}}C^{2}{\left(P\xi \right)}^{2}\left(\ln\left(\frac{{\left(P\;\xi \right)}^{2}}{1+{\left(P\;\xi \right)}^{2}}\right)-\;\ln{\left(\frac{\varepsilon }{C}\right)}^{2}\right)\right). \end{array}$$

As *P* = *P*(*r*_sky_) it is $\frac{\partial \;g\left(P\right)}{\partial r_{\mathrm{sky}}}=\frac{\partial \;g\left(P\right)}{\partial P}\frac{\partial P}{\partial r_{\mathrm{sky}}}$ with $\frac{\partial P}{\partial r_{\mathrm{sky}}}=\frac{\left(\xi r_{\mathrm{sky}}+1\right)}{\xi \;r_{d}}e^{\xi \left(r_{\mathrm{sky}}-r_{d}\right)}=P\;\left(\xi +\frac{1}{r_{\mathrm{sky}}}\right)$. Hence, $\frac{\partial \;g\left(P\right)}{\partial r_{\mathrm{sky}}}=\frac{\partial \;g\left(P\right)}{\partial P}\left(P\left(\xi +\frac{1}{r_{\mathrm{sky}}}\right)\right)=0$. But $\;\xi +\frac{1}{r_{\mathrm{sky}}}\neq 0$ always for 0 ≤ *r*_sky_ ≤ *r_d_* and *P* = 0 for the trivial case *r*_sky_ = 0 so that the minimization condition is rewritten in the form:

(22a) $$\frac{\partial \;g\left(P\right)}{\partial P}=0.$$

The minimization condition yields a fifth-degree equation in *P*. This gives two physical solutions, the smallest one corresponding to the equilibrium skyrmion radius *r*_0 sky_ at the minimum skyrmion energy (*∂*^2^*E*/*∂r*_sky_^2^ > 0) and the larger one to the skyrmion radius at the energy maximum ((*∂*^2^*E*/*∂r*_sky_^2^ < 0) and three unphysical solution.

To calculate *r*_0 sky_ (defined as *m_z_* (*r*_0 sky_) = 0) from Equation (22a), some reasonable approximations have been made in order to get a simple analytical expression of the equilibrium skyrmion radius. First, all terms of the form 1 + powers of (*Pξ*) were approximated to 1, taking into account that in correspondence of the energy minimum the value of the skyrmion radius is such that *P→*0 *(B << C, B→*0*)*. Second, arccot[*Pξ*] was expanded to the zero-order, viz. arccot[*Pξ*] ≈ *π*/2, introducing a coefficient Δ = *π*/2 + *η* (*T*) with Δ = Δ (*T*). Here, *η* is a very small coefficient (see Section 3 for the numerical details) depending on *T* and decreasing approximately with the same rate as *ξ* (*T*) that includes the effects of the other approximations and that, for the sake of simplicity, was added, as a small correction, to *π*/2. For the minimization calculation at *H*_ext_ ≠ 0, the term proportional to $\ln\left(\frac{{\left(P\;\xi \right)}^{2}}{1+{\left(P\;\xi \right)}^{2}}\right)-\;\ln{\left(\frac{\varepsilon }{C}\right)}^{2}$ that appears in the expression of the derivative *∂g*(*P*)/*∂P* has been neglected, assuming that *B* = *C P* has the same order of infinitesimal of *ε* (*ε* = 0.001) for the values of *B* crucial for searching the equilibrium radius ranging between 0 and those corresponding to the equilibrium radius itself that are still very small if compared to *C*. Owing to these approximations, it is possible to extract the equilibrium skyrmion radius from the following first degree equation in *B* via *B* = *P C*:

(22b) $$\left(8\beta \pm \frac{2\gamma }{\xi^{3}}-2\alpha \xi \Delta +\xi^{2}\right)B-\alpha \Delta =0.$$

The equilibrium skyrmion radius in dimensionless units is calculated from *B*_0_ = *r*_0 sky_ exp*^(ξ r^*_0sky_^)^.

In particular, *r*_0sky_ in the absence of an external magnetic bias field, *γ* = 0, reads:

(22c) $$r_{0\;\mathrm{sky}}≃\frac{1}{\xi }W\left(\frac{\alpha \;\xi \;\Delta }{8\beta \;-2\alpha \xi \Delta +\xi^{2}}\right).$$

Instead, *r*_0sky_ when the skyrmion is subjected to a perpendicular external magnetic bias field, *γ* ≠ 0, takes the form:

(22d) $$r_{0\;\mathrm{sky}}≃\frac{1}{\xi }W\left(\frac{\alpha \;\xi \;\Delta }{8\beta \;\pm \frac{2\gamma }{\xi^{3}}-2\alpha \xi \Delta +\xi^{2}}\right)$$

with *α* = *α* (*T*), *β* = *β* (*T*), *γ* = *γ* (*T*), and Δ = Δ (*T*). The skyrmion radius is proportional to the Lambert function *W*(*y*) depending on the magnetic parameters that are, in turn, scaled as a function of *T* and the parameter *ξ*. Here, ±2*γ* refers to *m_z_* = −1 (*m_z_* = +1) in the core center (*r* = 0) and *m*_z_ = +1 (*m_z_* = −1) at the skyrmion border (corresponding to *S* = −1 (*S* = +1)). Moreover, 8*β* −2*αξ* Δ ± 2*γ/ξ*^3^ +*ξ*^2^ > 0 for the scaled parameters used at every *T* and for any external magnetic field investigated ensuring the positivity of the argument *y* of *W*(*y*). This result is general and valid for both magnetization textures.

Note that the determination of the skyrmion size as a function of the magnetic parameters has been the subject of several investigations [45,46,47]. The expressions of *r*_0 sky_ of Equations (22c) and (22d) have some similarities with other expressions of the equilibrium skyrmion radius recently derived in the literature from the minimization of the skyrmion energy, where *r*_0 sky_ is a function of the magnetic parameters appearing in a fractional form [45,46]. However, note that, in the present approach, the equilibrium magnetization distribution is an accurate 2D distribution that recovers for *Q* = 1 and *D* = 0 the Belavin–Polyakov soliton solution, while in those studies the magnetization distribution had the form of an approximated cosine-like variation or resulted from a 360° domain-wall distribution.

According to Equations (22c) and (22d), as *T* increases, *ξ* decreases for the scaled parameters used and *W(x)* increases leading to an increase of *r*_0sky_ for any fixed external field amplitude and for any magnetization texture. The equilibrium skyrmion radius in dimensional units reads *R*_0 sky_ = *r*_0 sky_ *l*_exch_ at any *T* and for any external magnetic field and the equilibrium skyrmion diameter is *D*_0 sky_ = 2 *R*_0 sky_. For the case considered in the numerical calculations (Nèel skyrmion with *S* = −1 and radially outward magnetization, *χ* = +1), it has been found that this behavior is confirmed. This trend characterizing the equilibrium skyrmion diameter as a function of *T* is indeed consistent with the one obtained calculating the skyrmion energy via the numerical evaluation of the integrals and of the equilibrium skyrmion diameter (for the comparison of the equilibrium skyrmion diameter calculated by means of Equations (22c) and (22d) and numerically, see Section 3.1). Because of the invariance of the DMI energy, this trend occurs also for a Nèel skyrmion with *S* = +1 and radially inward magnetization, *χ* = −1. However, due to the generality of Equations (22c) and (22d), this behavior characterizes *r*_0sky_ also for a Bloch skyrmion (either *S* = +1 and counter-clockwise chirality, *χ* = +1, or *S* = −1 and clockwise chirality, *χ* = −1).

Moreover, at fixed *T*, *r*_0sky_ has different trends with increasing *H*_ext_ (either positively or negatively). In particular, for a Néel or a Bloch skyrmion with *S* = −1 and *χ* = +1 and *H*_ext_ > 0 (*H*_ext_ < 0), *r*_0sky_ reduces (increases) with increasing the positive (negative) amplitude of the external magnetic field leading to a positive (negative) increase of *γ*, *γ* > 0 (*γ* < 0), confirming recent micromagnetic and numerical calculations [43].

Finally, according to Equations (22c) and (22d), at fixed magnetic parameters for a given *T*, the equilibrium skyrmion radius does not have an explicit dependence on the dot radius. By means of a comparison with the equilibrium radius calculated from the numerical minimization of the skyrmion energy it has been found that, for the magnetic parameters used, this is approximately valid for a dot radius *R*_d_ > 50 nm. For *R*_d_ ≤ 50 nm *R*_0 sky_ depends on the dot radius and reduces with decreasing *R*_d_ as found by Tejo et al. [44] so that, in this regime Equations (22c) and (22d) are not anymore valid.

### 2.3. Statistical Thermodynamic Properties of Skyrmion Diameters Population

In this section the statistical thermodynamics aspects of chiral magnetic skyrmions at equilibrium are investigated taking into account the strict analogy with the particles behavior in an ideal gas. In particular, key quantities to understand their thermodynamic behavior such as the partition function and the free energy are calculated within a microcanonical ensemble. Finally, the pressure is determined and an equation of state linking pressure, volume, and temperature is proposed.

#### 2.3.1. Partition Function and Free Energy of a Skyrmion Diameters Population within a Microcanonical Ensemble

Let us calculate the partition function and the free energy of a skyrmion diameters population within a microcanonical ensemble. Very recently, it has been shown that, starting from a canonical ensemble, a skyrmion diameters population can be approximately described within a microcanonical ensemble due to the small fluctuations of the energy around the average energy. To calculate the partition function, the expression of the configurational entropy at thermodynamic equilibrium resulting from an average over the 3D equilibrium Maxwell–Boltzmann distribution of skyrmion diameters is recalled [39]:

(23) $$S≃k_{B}\left[\ln\left(\frac{{\left(k_{B}T\right)}^{\frac{3}{2}}+2{\left(k_{B}T\right)}^{\frac{1}{2}}a\;<D_{\mathrm{sky}}{>}^{2}}{a^{\frac{3}{2}}<D_{\mathrm{sky}}{>}^{2}t}\right)+\frac{1}{2}\left(\frac{3k_{B}T+2a\;<D_{\mathrm{sky}}{>}^{2}}{k_{B}T+2a\;<D_{\mathrm{sky}}{>}^{2}}\right)\right]+S_{0}$$

with *a* = *a*(*T*) a parameter proportional to the parabola curvature, <*D*_sky_> = <*D*_sky_(*T*)> the average skyrmion diameter, and *S*_0_ = *k*_B_ (2 ln 2+1/2 ln *π*). <*D*_sky_> was calculated as a spatial integral of *D*_sky_ over the 3D Maxwell–Boltzmann distribution [39]. The reason for performing the analysis in a 3D space instead of a 2D (the magnetization distribution is indeed planar) results from the observation, via micromagnetic simulations, that the thickness of the nanodot (even though less than 1 nm) can be important to establish the behavior of the diameters distribution. Strictly speaking, Equation (23) was derived for a Nèel skyrmion with *S* = −1. However, as already outlined in [39], this expression can be considered valid also for a Nèel skyrmion with skyrmion number *S* = +1 and applies also to the Bloch skyrmion with *S* = ±1. Indeed, the energy takes the same form for any magnetization texture and its trend in the vicinity of the minimum can be approximated by a quadratic dependence on the skyrmion diameter.

Note that, as for the ideal gas, configurational entropy expressed by Equation (23) fulfills the requirement of additivity (entropy is indeed an extensive thermodynamic quantity), is consistent with the definition of temperature and with the second principle of thermodynamics, and with the adiabatic invariance for slow changes occurring in the system. On the other hand, due to its classical derivation, *S* does not fulfil the third principle of thermodynamics or Nernst principle according to which *S* = 0 J/K for *T* = 0 K, but *S* → −∞ as *T*→0 K, showing a similar behavior to that of the Sackur–Tetrode equation expressing the entropy for an ideal gas as a function of *T* [39].

Equating the calculated configurational entropy to *S* = *k*_B_ ln*W* representing the general entropy definition valid for a microcanonical ensemble, *W* can be determined. In statistical mechanics, the quantity *W* represents the number of microscopic possibilities to realize the macroscopic state. More specifically, *W* can be regarded as the statistical multiplicity of the energy level having value <*E*> or as the degeneracy of the ground state of the skyrmions population having average energy <*E*>. One gets:

(24) $$W\;≃K\;e^{\;-\;\frac{2a\;<D_{\mathrm{sky}}{>}^{2}}{2a\;<D_{\mathrm{sky}}{>}^{2}+k_{B}T}}\;\frac{2a\;<D_{\mathrm{sky}}{>}^{2}{\left(k_{B}T\right)}^{\frac{1}{2}}+{\left(k_{B}T\right)}^{\frac{3}{2}}}{a^{\frac{3}{2}}<D_{\mathrm{sky}}{>}^{2}}\;$$

where *K* = 4 √*π e*^3/2^/*t* is a constant at fixed dot thickness *t*. The statistical multiplicity of a skyrmion diameters population depends not only on *T* but also on the geometric and magnetic parameters of the magnetic system that, in turn, have an intrinsic dependence on *T*.

It can be noted that *W* = 0 for *T* = 0 K: at the absolute zero the ground state is no longer degenerate. With increasing *T* there is an enhancement of the degeneracy. This means that the higher the temperature the greater the probability to populate the ground state levels of skyrmion diameters population.

In [39], it has been also shown that the partition function of the skyrmion diameters population within a canonical ensemble can be approximated by the one of the microcanonical ensemble that in the discrete limit reads *Z* ≈ *W e* ^−^^<*E*>/(*k*^_B_*^T^*^)^. Hence, replacing *W* given in Equation (24), the explicit expression of the partition function within a microcanonical ensemble is obtained:

(25) $$Z≅\;K\frac{e^{-\;\left(\frac{2a\;<D_{\mathrm{sky}}{>}^{2}}{2a\;<D_{\mathrm{sky}}{>}^{2}+k_{B}T}\right)\;}e^{\;-\;\frac{5/2k_{B}T+2\;a{\left(D_{0\mathrm{sky}}\right)}^{2}}{2\;k_{B}T}\;}}{a^{\frac{3}{2}}<D_{\mathrm{sky}}{>}^{2}}\left(2a\;<D_{\mathrm{sky}}{>}^{2}{\left(k_{B}T\right)}^{\frac{1}{2}}+{\left(k_{B}T\right)}^{\frac{3}{2}}\right)$$

where <*E*> ≅ (5 *k*_B_*T*+2*a*(*D*_0 sky_)^2^)/2 is the average energy calculated in [39] as <*E*> ≅ *a* <*D*_sky_^2^> and *D*_0 sky_ = *D*_0 sky_ (*T*) is the equilibrium skyrmion diameter with *D*_0 sky_ ≅ <*D*_sky_>, especially at low *T*. Looking at Equation (25) it can be observed that the partition function of a skyrmion diameters population depends on fractional powers of *T*. However, there is a combined dependence on *T*^1/2^ and *T*^3/2^ if compared to the partition function for an ideal gas whose particles follow a 3D MB distribution where there is only a *T*^3/2^ dependence. Moreover, the fractional dependence on *T* appearing in the rational fraction is weighted by an exponential term that also depends on *T*. This exponential dependence differentiates the partition function *Z* of a skyrmions population from that of the particles of a 3D ideal gas.

Similarly to the configurational entropy, the partition function has a combined dependence on geometric and magnetic parameters. Taking into account the relation between the Helmholtz free energy *F* and the partition function of the microcanonical ensemble expressed as *F* = −*k*_B_*T* ln*Z*, the relation *F* ≅ <*E*> − *T S* can be easily found, with *F* = *F*(*T*) for every *T* (the symbol ≅ results from the approximated form of *Z*), the well-known thermodynamic relation generally used for ferromagnets. For this case, because of the definition of *S* as an expectation value, *F*, while depending on *T*, is regarded as an average free energy. Note that the Zeeman energy is included in <*E*> and, for this reason, still one deals with the Helmholtz free energy *F*. However, if this contribution is thought as separated from the other skyrmion energy contributions to the average energy one would deal with the Gibbs free energy *G*. Within this framework, the two approaches are equivalent.

#### 2.3.2. Skyrmion Diameters Population Pressure and Equation of State

From the free energy, it is possible to derive, in complete analogy with the pressure exerted by the particles of a gas on the walls of a container, the pressure *p* of the skyrmion diameters population.

Taking into account the infinitesimal relation *dF* = − *S dT* − *p dV* it is *p* = − (*∂F*/*∂V*)*_T_*. In explicit form:

(26) $$p=-{\left(\frac{\partial F}{\partial V}\right)}_{T}.$$

Equation (26) gives the pressure generated by skyrmion diameters population at a fixed temperature *T*. If a container filled with an ideal gas is expanded instantaneously, the temperature of the gas does not change at all. Analogously, if the skyrmion size is allowed to fluctuate instantaneously, the skyrmion diameter deviates from the average value and, as a result, the skyrmion volume fluctuates around its average value. This process occurs without affecting the temperature, as occurs for particles in an ideal gas.

To calculate the pressure, one could substitute either *F* ≅ <*E*> − *T S* or *F* = −*k*_B_ *T* ln *Z* in Equation (26). However, unlike for the case of an ideal gas, the thermodynamic variables <*E*>, *S*, and *Z* within this framework have a dependence not on the generic volume *V* but on the average skyrmion volume <*V*> with in addition <*V*> = <*V*(*T*)>, that is the dependence on *V* is mixed with that of *T*. Therefore, it is convenient to argue in terms of densities of thermodynamic variables. Let us introduce a free energy density *f* = *f*(*D*_sky_) dependent on skyrmion diameter with *f* = *e* − *T s*, where *e* = *e*(*D*_sky_) is the internal energy density (internal energy per unit volume *e* = *E*/*V*) and *s* = *s*(*D*_sky_) the configurational entropy density (configurational entropy per unit volume). These quantities are expressed taking into account the approximation on the skyrmion energy in the vicinity of the minimum via a quadratic dependence [39] on *D*_sky_, viz. *E* = *a* (*D*_sky_ − *D*_0sky_)^2^ used to calculate <*D*_sky_> and the skyrmion configurational entropy expressed in Equation (23). Note that, for the total computation of the energy, an energy shift of *b* = *E* (*D* = *D*_0 sky_) would be added to this expression that gives the energy in the minimum. This harmonic approximation is reasonable because the main contribution to the configurational entropy results from skyrmion diameters around the energy minimum. In particular:

*e* ≅ *a* (*D*_sky_ − <*D*_sky_>)^2^/*V* (27)

where *V* = ¼ *π D*_sky_^2^ is the skyrmion volume for a given *D*_sky_ and the symbol ≅ was introduced because, for consistency with the definition of entropy density (see below), *D*_0 sky_ has been replaced with <*D*_sky_>. For the scaled parameter used, this approximation holds in the region of metastability being the difference between <*D*_sky_> and *D*_0 sky_ less than 10 %. The volume *V* is at most equal to the dot volume *V* = ¼ *π D*_d_^2^, with *D*_d_ = 2 *R*_d_ the dot diameter.

Instead, the entropy density can be derived from the definition of the Boltzmann order function at equilibrium equivalent (apart from the sign) to the Shannon information entropy. First, the Gaussian diameters distribution or probability density appearing in the Boltzmann order function at equilibrium is considered, $f_{0}=N\;e^{\;-\;\frac{a\;{\left(\Delta D_{\mathrm{sky}}\right)}^{2}}{k_{B}T}\;}$, with Δ*D*_sky_ = *D*_sky_ − <*D*_sky_> expressing the deviation from the average skyrmion diameter at a given *T* and *N* the normalization constant. The normalization constant has the dimension of an inverse of a volume and is obtained by normalizing to one skyrmion having different sizes and diameters in different instants of time, giving rise to a skyrmion diameters population at each *T* in the range of temperatures corresponding to the metastable state, as shown by micromagnetic simulations [39]. Here, *f*_0_ has the meaning of a Gaussian probability density. In principle, one should normalize the Gaussian distribution in the usual way, performing a volume integration of *f*_0_. This results in a value of *N* that has an explicit dependence on *T*, on <*D*_sky_>, and on magnetic parameters through *a*, and is crucial for the calculation of the configurational entropy. However, to have a strict connection with the magnetic skyrmion volume *V* = *V*(*D*_sky_) that strictly depends on the skyrmion diameter *D*_sky_ for each *T*, one takes, without loss of generality, *C* =1/*V*. Hence, one writes $f_{0}=\frac{1}{V}e^{\;-\;\frac{a\;{\left(\Delta D_{\mathrm{sky}}\right)}^{2}}{k_{B}T}\;}$. Note that *f*_0_ is not anymore, strictly speaking, a Gaussian distribution because *V*∝ (*D*_sky_)^2^ leading to a singularity of *f*_0_ for vanishing *D*_sky_. However, for diameters different from zero its trend is almost superimposable to that of the corresponding Gaussian, with a slight downshift of the maximum (with respect to the maximum for *D*_sky_ = <*D*_sky_> of the Gaussian) that increases with increasing temperature and a slight variation of the standard deviation and of the full width at half maximum.

In view of the above arguments the entropy density *s* = −*k*_B_ *f*_0_ ln *f*_0_ can be written in the form:

(28) $$s=-k_{B}\frac{1}{V}e^{-\;\frac{a\;{\left(\Delta D_{\mathrm{sky}}\right)}^{2}}{k_{B}T}\;}\ln\frac{<V>}{V}+\frac{1}{V}e^{\;-\;\frac{a\;{\left(\Delta D_{\mathrm{sky}}\right)}^{2}}{k_{B}T}\;}\frac{a\;{\left(\Delta D_{\mathrm{sky}}\right)}^{2}}{T}.$$

This expression can be derived from the integrand of the Boltzmann order function *H*_0_ defined in the continuum limit as an integral over spatial coordinates and evaluated at equilibrium replacing the normalization constant *C* obtained integrating the Gaussian distribution over the spatial coordinates [39] with the volume *V* of the skyrmion and rescaling ln*f*_0_ to ln(*f*_0_ <*V*>) for dimensional reasons. According to these definitions, the pressure is rewritten in the form:

(29a) $$p=\;-<V>\left[{\left(\frac{\partial \;e}{\partial V}\right)}_{T}-T\;{\left(\frac{\partial \;s}{\partial V}\right)}_{T}\right]$$

where <*V*> ≅ ¼ *π* <*D*_sky_>^2^ is the average volume according to the approximation <*D*_sky_^2^> ≈ <*D*_sky_>^2^ that holds for the range of temperatures considered. Indicating the source of pressure having energy nature with *p_E_* = −<*V*> (*∂e*/*∂V*)*_T_*, one obtains substituting Equation (27):

(29b) $$p_{E}≃\;<V>\frac{a\;{\left(\Delta D_{\mathrm{sky}}\right)}^{2}}{V^{2}}.$$

One notes that this pressure contribution is always positive and, at fixed *a*, it decreases with increasing *V*.

Labeling with *p_S_* = <*V*> *T* (*∂s*/*∂V*)*_T_* the source of pressure having entropic nature, via Equation (28), yields:

(29c) $$p_{S}=\frac{<V>}{V^{2}}e^{-\;\frac{a\;{\left(\Delta D_{\mathrm{sky}}\right)}^{2}}{k_{B}\;T}\;}\left[k_{B}T\left(\ln\frac{<V>}{V}+1\right)-a\;{\left(\;\Delta D_{\mathrm{sky}}\right)}^{2}\right]$$

with <*V*>/ *V* = <*D*_sky_>^2^ */D*_sky_^2^. This contribution is negative for low volumes, positive for intermediate volumes close to the average volume, and again positive for high volumes bigger than the average volume and is thus responsible for the oscillatory behavior of the pressure curve, especially the one present at high volumes (see Section 3.4). Moreover, *p_S_* is about one order of magnitude less than *p_E_*.

Pressure *p* is obtained by summing the two contributions of Equations (29b) and (29c), viz. *p* = *p_S_* + *p_E_*:

(29d) $$p≃\frac{<V>}{V^{2}}\left[e^{-\;\frac{a\;{\left(\Delta D_{\mathrm{sky}}\right)}^{2}}{k_{B}\;T}\;}\left(\left(\ln\frac{<V>}{V}+1\right)\right)k_{B}T+a\;{\left(\Delta D_{\mathrm{sky}}\right)}^{2}\left(1-e^{-\;\frac{a\;{\left(\Delta D_{\mathrm{sky}}\right)}^{2}}{k_{B}T}\;}\right)\right]\;$$

where the symbol ≃ results from the approximated energy contribution to the pressure. Straightforwardly, *p* written in terms of dimensionless variables reads:

(29e) $$p\;≃\;\frac{1}{V}\left[\left(f\left(\Delta D_{\mathrm{sky}}\right)\;\frac{g\left(v\right)}{v}\right)\;k_{B}T\;+\frac{1}{v}f_{c}\left(\Delta D_{\mathrm{sky}}\right)a\;{\left(\Delta D_{\mathrm{sky}}\right)}^{2}\right]$$

where *f*(Δ*D*_sky_>) = *e* ^-*a* (Δ*D*^_sky_^)^^2/*k*^_B_*^T^* and *g*(*v*) = ln(1/*v*) +1, with *v* = *V*/<*V*> (*v = D*_sky_^2^ /<*D*_sky_>^2^) a dimensionless variable and *f*_c_(Δ*D*_sky_>) = 1 − *e* ^-*a* (Δ*D*^_sky_^)^^2/*k*^_B_*^T^*. At fixed *T*, pressure reduces with increasing *V* but exhibits a superimposed fluctuating behavior for *V* > <*V*> for each *T* (for the details of the numerical calculations, see Section 3) due to the statistical dependence on the skyrmion diameters contained in the coefficient of *k*_B_*T* and in the second term on the second member. This statistical aspect differentiates the skyrmion population behavior from that of pressure exerted on the walls of a box by the particles of an ideal gas that has a monotonic behavior as a function of *V* for fixed *T*.

By inspecting Equations (29b) and (29c), one notes that both sources of pressure depend on the confining skyrmion potential or skyrmion energy whose curvature in the vicinity of the absolute minimum is determined by the coefficient *a*, in turn depending on the magnetostatic field and the exchange interactions. From Equation (29e) one gets:

(30) $$p\;V≃\;\left(f\left(\Delta D_{\mathrm{sky}}\right)\frac{g\left(v\right)}{v}\right)k_{B}T\;+\frac{1}{v}f_{c}\left(\Delta D_{\mathrm{sky}}\right)a\;{\left(\Delta D_{\mathrm{sky}}\right)}^{2}.$$

Equation (30) is the equation of state for a skyrmion diameters population and is the main result of this study. The second member contains a term proportional to *T* weighted by a coefficient in turn depending on *f*(Δ*D*_sky_>) and a term proportional to the square deviation of *D*_sky_ from <*D*_sky_> in turn weighted by *f*_c_(Δ*D*_sky_>). This equation describes any single chiral skyrmion diameters population independently of its magnetization texture and ferromagnetic material considered.

Straightforwardly, for *D*_sky_ = <*D*_sky_> it is *p* =*k*_B_*T/V*, the pressure exerted by a single particle (*N* = 1) on the walls of the container of volume <*V*> = *V* that, in turn, yields *pV* = *k*_B_*T*, the equation of state for an ideal gas for *N* = 1.

It is interesting to derive the asymptotic behavior of the equation of state in the limit for *a*(*D*_sky_ − <*D*_sky_>)^2^ << *k*_B_*T* for small fluctuations of diameters around the average value <*D*_sky_>. This regime is named the small fluctuations regime. This occurs at low temperatures, especially in the presence of an applied bias field characterized by sharp Gaussian distribution [35].

For *a*(Δ*D*_sky_)^2^ << *k*_B_*T*, the exponential *e*^−*a* (Δ*D*^_sky_^)^^2/*k*^_B_*^T^* in Equation (30) is expanded to the first-order in (Δ*D*_sky_)^2^ and the fourth-order term *a*^2^(Δ<*D*_sky_>)^4^/*k*_B_*T* is neglected being *a*^2^(Δ<*D*_sky_>)^4^ << *k*_B_*T*, yielding:

(31) $$pV+\frac{g\left(v\right)}{v}a\;{\left(\Delta D_{\mathrm{sky}}\right)}^{2}≃\frac{g\left(v\right)}{v}k_{B}T.$$

Equation (31) is the equation of state of a skyrmion diameters population in the small fluctuations regime and is valid for values of *D*_sky_ very close to <*D*_sky_>. For *D*_sky_ = <*D*_sky_> one gets *g*(*v*) = 1 and *v* = 1 so that again the ideal gas law *pV* = *k*_B_*T* for *N* = 1 is obtained.

## 3. Results and Discussion

In this section the main numerical results are discussed. The numerical calculations were carried out for an outwardly Neel skyrmion with *S* = −1 (magnetization in the core center along −*z*) hosted in a Co dot of *R*_d_ = 200 nm and *t* = 0.8 nm using the following magnetic parameters at *T* = 0 K: saturation magnetization *M*_s_ = 600 KA/m, exchange stiffness constant *A* = 20 pJ/m, IDMI parameter *D* = 2.0 mJ/m^2^, *K*_u_ = 0.6 MJ/m^3^ (for example Co), and the scaled values at *T* ≠ 0 according to the scaling laws expressed in Section 2 [35]. However, note that the model can be numerically applied to other magnetization textures present in ultrathin cylindrical dots. In Table 1 the values of *a* and <*D*_sky_> used in the numerical calculations presented in this Section are summarized [39].

The values of the equilibrium diameters for every temperature and external bias field are listed in Table 2.

### 3.1. Numerical and Analytical Equilibrium Diameters: A Comparison

**Figure 2:**
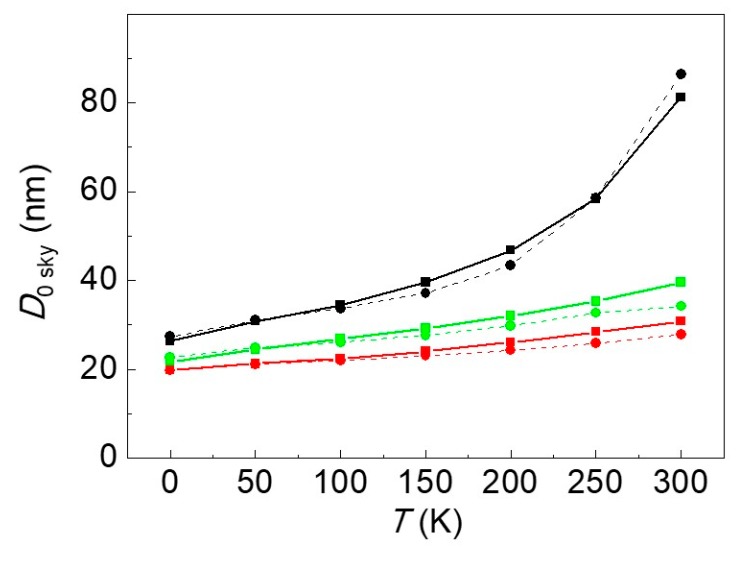
Comparison between *D*_sky_ obtained numerically (solid lines with squares) and *D*_sky_ calculated according to Equations (22c) and (22d) (dashed lines with circles). Black lines: *D*_sky_ at *μ*_0_*H*_ext_ = 0 mT. Green lines: *D*_sky_ at *μ*_0_*H*_ext_ = 25 mT. Red lines: *D*_sky_ at *μ*_0_*H*_ext_ = 50 mT.

In this subsection the equilibrium diameters calculated by means of Equations (22c) and (22d) via *D*_0sky_ = 2*R*_0sky_ are compared with the ones determined numerically. The numerical calculations were performed minimizing the skyrmion energy of Equation (7) using Equations (A6)–(A14) and determining the equilibrium radius as a function of temperature and for different bias external fields. The results of this comparison are shown in Figure 2. The overall agreement is very good for the whole range of temperatures investigated, with a more pronounced discrepancy, especially at high temperatures.

In the numerical calculations, the values of the coefficient *η* appearing in Δ = *π*/2 + *η* of Equations (22b), (22c) and (22d) summarized in Table 3 were used. The coefficient *η* was fitted at *T* = 0 K to the value of *D*_0 sky_ evaluated numerically for three different amplitudes of the external field and linearly reduces with *T* with the same rate of decrease of *ξ* with *T*.

These results are easily generalizable to the case with skyrmion number *S* = +1 and other magnetization skyrmion textures (e.g., to a Bloch skyrmion) in the region of metastability.

### 3.2. Energy of the Skyrmion State and of Perpendicular Uniform State: A Comparison

**Figure 3:**
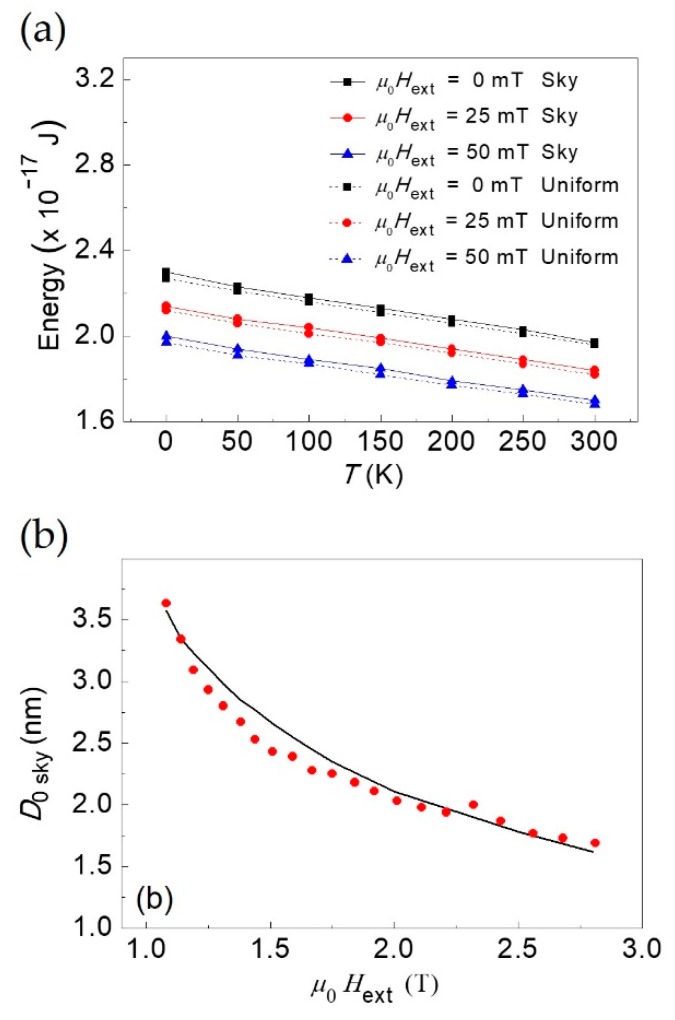
(**a**) Skyrmion state energy minimum compared to uniform state energy minimum for *μ*_0_*H*_ext_ = 0 mT and in the presence of an external field of amplitude *μ*_0_*H*_ext_ = 25 mT and *μ*_0_*H*_ext_ = 50 mT, respectively. Continuous lines: skyrmion energy minimum vs. *T*. Dashed lines: corresponding energy of the uniform state for the same external bias field amplitudes; (**b**) Equilibrium skyrmion diameter vs. the external bias field. Continuous black line: analytical calculation from the minimization of the skyrmion energy. Red circles: experimental data obtained with spin-polarized scanning tunnel microscopy [48] at *T* = 4.2 K.

In Figure 3a, the skyrmion energy evaluated at the energy minimum as a function of temperature in the skyrmion state in the region of metastability is displayed and compared to the skyrmion energy of the ideal uniform state in the presence of an external magnetic field aligned along +*z* (magnetization distribution along +*z* in the whole dot and parallel to the external magnetic field).

The skyrmion energy was calculated by numerically integrating Equation (7) and expressing the total skyrmion energy using Equations (A6)–(A14). Instead, the energy of the ideal uniform state along +*z* is expressed as:

(32) Euniform=(12μ0Ms2−μ0MsHext)V.

As expected, the energy of the uniform state is downshifted with respect to that of the skyrmion state at each *T* [44]. Between the two minima, there is an energy barrier, mainly due to the ferromagnetic exchange and the *i*-DMI contributions, and the energy minimum of the uniform state is lower with respect to that of the skyrmion state. This behavior marks the metastability of the skyrmion state. In particular, with increasing temperature, the two minima tend to merge. To benchmark the model, the equilibrium diameters computed as a function of the external bias field (continuous black line) have been compared with the ones obtained with spin-polarized scanning tunnel microscopy (red circles) and the result of the comparison is shown in Figure 3b. Also in this case, the equilibrium diameters were calculated by numerically minimizing the skyrmion energy of Equation (7) using Equations (A6)–(A14). The geometric and magnetic parameters used in the calculations are: *R*_d_ = 25 nm, *t* = 0.408 nm, *A* = 2.0 pJ/m, *D* = 3.9 mJ/m^2^, *K* = 2.5 MJ/m^3^, and *M*_s_ = 1.1 MA/m. The magnetic parameters are typical values for thin-film ferromagnetic systems, such as the bilayer of PdFe on Ir (111) single crystal substrate [48]. Note that in that case the external field is anti-parallel to the magnetization at the center of the skyrmion core but with *m*_z_ (*r* = 0) = +1 and **H**_ext_ along −*z*. This configuration is equivalent to the one studied in this work with *m*_z_ (*r* = 0) = −1 and **H**_ext_ along +*z*. The agreement is very good for the whole interval of external bias fields studied.

### 3.3. Microcanonical Partition Function and Free Energy of the Skyrmions Population

**Figure 4:**
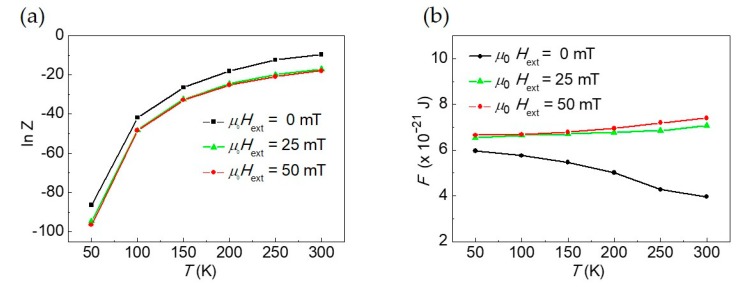
(**a**) Partition function vs. *T* at *μ*_0_ *H* = 0 mT (black line with squares), *μ*_0_ *H* = 25 mT (green line with up triangles), and *μ*_0_ *H* = 50 mT (red line with diamonds); (**b**) As in panel (**a**) but for the Helmholtz free energy *F*.

The partition function and the Helmholtz free energy of the skyrmion diameters population studied within a microcanonical ensemble are shown in Figure 4. The partition function was calculated according to Equation (25) by varying the temperature *T* at fixed external magnetic field. The Helmholtz free energy is computed via the relation *F* = −*k*_B_*T* ln*Z*. The values of the average skyrmion diameters entering in the expressions of Z and *F* are the ones listed in Table 1 for the three different amplitudes of the external bias field, *μ*_0_*H*_ext_ = 0 mT, *μ*_0_*H*_ext_ = 25 mT, and *μ*_0_*H*_ext_ = 50 mT.

Looking at Figure 4a, ln *Z* increases with increasing *T*, reflecting the increase of *Z* with *T*, a number ranging between 0 and 1. Instead, one notes that the effect of the external magnetic field is to reduce *Z*. This can be understood taking into account the physical meaning of the partition function in statistical thermodynamics. *Z* encodes how the probabilities are partitioned among the different microstates, based on their individual energies. The partition function *Z* for *T*→0 K vanishes and, for *T* ≠ 0 K, increases with *T*. Due to the disordering effect introduced by the temperature on the thermodynamic system, with increasing *T* the partitioning probability among different microstates of the skyrmion diameters population increases. The ordering effect of the external bias field as in the case of configurational entropy [39] partially contrasts with the disordering effect of *T* attenuating the increase of *Z* with *T*.

On the other hand, the trend of the Helmholtz free energy (with *F* > 0) as a function of *T* in the presence of an external bias field is almost constant while, in its absence, *F* decreases with *T*. *F* merges asymptotically towards a value of about 6 × 10^−21^ J as *T*→0 K (see Figure 4b), masking almost completely the effect of *H*_ext_. The behavior of the Helmholtz free energy *F* as a function of *T* is different with respect to the one of <*E*> that increases with *T*.

The effect of the external magnetic field leading to a downshift of *Z* and *F* would be similar considering the case with **H**_ext_ applied along –*z* and *m_z_*(*r* = 0) = +1 (*S* = +1). Instead, an opposite behavior leading to an upshift of the same thermodynamic quantities in the cases of an applied field parallel to *m_z_* (either along −*z* (*m_z_*(*r* = 0) = −1 and *S* = −1) or along +*z* (*m_z_*(*r* = 0) = +1 and *S* = +1) should be expected.

### 3.4. Skyrmions Population Pressure and Equation of State

**Figure 5:**
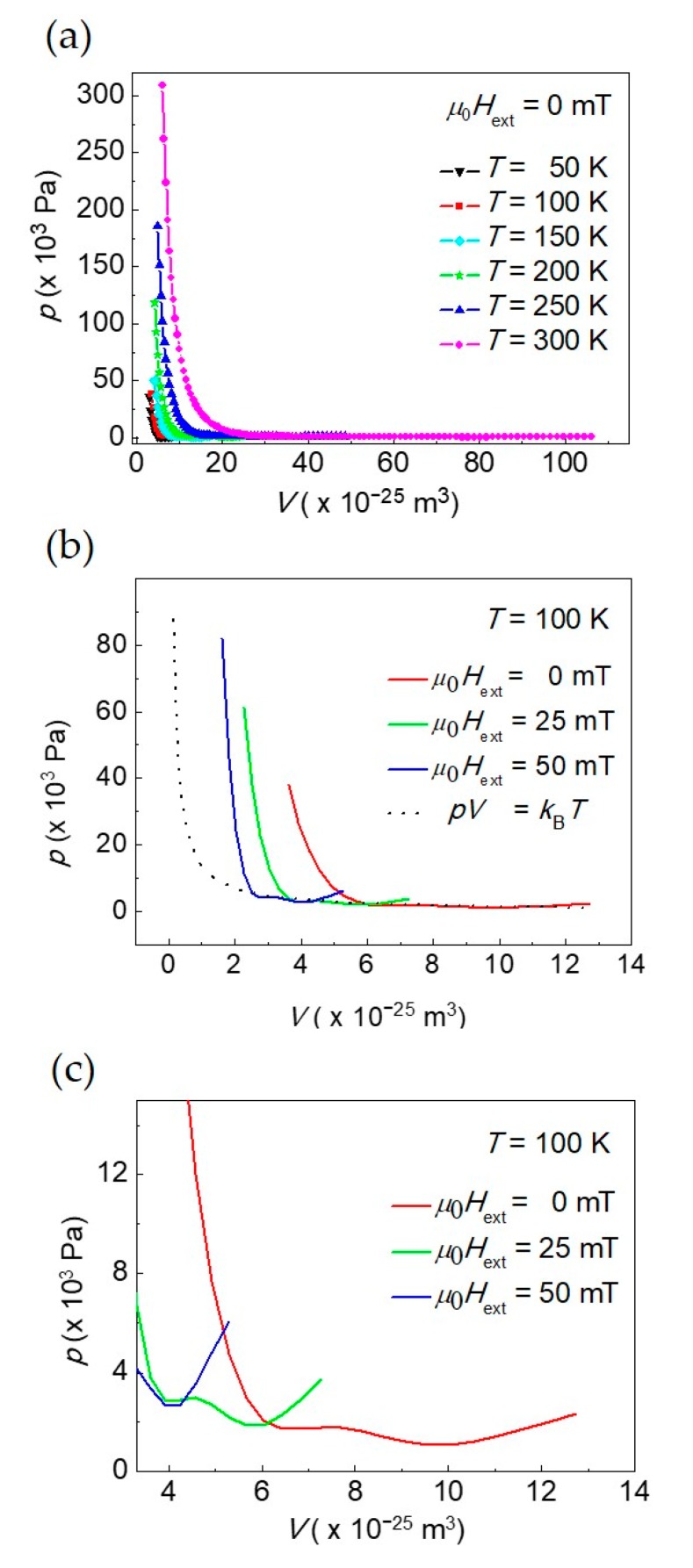
(**a**) Solid lines with symbols: pressure of the skyrmions population calculated according to Equation (29e) as a function of the skyrmion volume at different temperatures in zero applied magnetic field (black line with down triangles: *T* = 50 K, red line with squares: *T* = 100 K, cyan line with diamonds: *T* = 150 K, green line with stars: *T* = 200 K, blue line with up-triangles: *T* = 250 K, magenta line with circles: *T* = 300 K). (**b**) Solid lines: as in panel (**a**) but for *T* = 100 K and *μ*_0_*H*_ext_ = 0 mT (red line), *μ*_0_*H*_ext_ = 25 mT (green line), *μ*_0_*H*_ext_ = 50 mT (blue line). Dotted line: pressure *p* = 1/*V* k_B_ *T* according to the ideal gas law for *N* = 1. (**c**) Pressure of the skyrmions population as a function of the skyrmion volume for *T* = 100 K and *μ*_0_*H*_ext_ = 0 mT (red line), *μ*_0_*H*_ext_ =25 mT, *μ*_0_*H*_ext_ =50 mT for volumes *V* > <*V>*.

The pressure of the skyrmions population as a function of the volume *V* of the skyrmion diameters population is shown in Figure 5 at fixed *T*. For each temperature *T* at fixed external bias field, volumes considered correspond to the diameters belonging to the range of about ±3*σ_<D_*_sky>_, where *σ_<D_*_sky>_ is the standard deviation of the Gaussian distribution centered at the average diameter *<D*_sky_>. In Figure 5a, pressure *p* vs. volume *V* in the absence of an external bias field at different temperatures (isotherms) is displayed. *p* was determined by means of Equation (29e) and its calculation takes into account the Gaussian distribution of skyrmion diameters fluctuations from *<D*_sky_> at each temperature and external bias field, with *<D*_sky_> given in Table 1 and the volume approximately computed as *V* ≈ ¼ *π* < *D*_sky_>^2^.

The general trend at all temperatures is the dramatic increase of *p* with decreasing *V* below a given volume, depending on the temperature *T*. This means that, for a skyrmion of reduced size, the pressure exerted on the region of the ferromagnet just outside the skyrmion core is very high if compared to a skyrmion of intermediate size. Moreover, at fixed volume, pressure reduces with reducing the skyrmion temperature in a way similar to that of the particles of an ideal gas with decreasing *T*.

Looking at Figure 5b, it can be observed that the pressure curve (depicted for *T* = 100 K) in the presence of an external magnetic field along −*z* that is anti-parallel to the static magnetization at the core center (*m_z_*(*r* =0) = +1) at fixed volume shifts towards lower values so that, at a given value of *V*, the effect of a bias field is to reduce pressure in a way similar to the reduction of the configurational entropy [39]. This trend is also typical for other temperatures and higher external magnetic fields. The skyrmion pressure curves intersect the curve *p* =*k*_B_*T*/*V* of the ideal gas at *V* = <*V*>: for *μ*_0_*H*_ext_ = 0 mT it is *V* = 7.78 × 10^−25^ m^3^, for *μ*_0_*H*_ext_ = 25 mT it is *V* = 4.70 × 10^−25^ m^3^ and for *μ*_0_*H*_ext_ = 50 mT it is *V* = 3.28 × 10^−25^ m^3^. One notes that the intersection with the ideal gas curve occurs at lower volumes in the presence of an external magnetic field. At intermediate and high volumes (*V* > <*V>*) there is an oscillatory behavior of the pressure curve superimposed to the monotonic behavior that is enhanced with increasing the amplitude of the external bias field (see Figure 5c). Similar conclusions are drawn for other values of temperature in the region of skyrmion metastability. Because of this oscillatory behavior introduced by the diameters distributions *f* and *f*_c_, unlike for the universal law for an ideal gas characterized by the universal constant *R* = *PV*/*T* at any *T*, the classical thermodynamics of a skyrmions population cannot be strictly characterized by a universal constant.

For values of *V* close to <*V*>, Equation (31) expressing pressure *P* in the small fluctuation regime can be applied. A similar shift towards lower values of the pressure curve would occur for the external magnetic field applied along −*z* and a Néel skyrmion with the magnetization at the centre of the core *m_z_*(*r* = 0) = +1 (*S* = +1). Instead, for the cases where the the effect is to increase the skyrmions population pressure at a given volume *V*.

These results can be easily generalized to other cases (e.g., external magnetic field is parallel to *m*_z_(*r* = 0) either along +*z* or along −*z*) and to Bloch skyrmions in the region of metastability, leading to conclusions about the qualitative trends of *p* obtained similar to the ones drawn for the case studied.

## 4. Conclusions

In this paper, the equilibrium statistical mechanics and thermodynamic properties of a skyrmion diameters population in an ultrathin cylindrical dot were investigated. An analytical expression of the internal skyrmion energy valid for any type of magnetization texture, consisting of a combination of transcendental functions, was obtained. From the minimization of the internal skyrmion energy it was found that the equilibrium diameter of a single chiral skyrmion, both in the hedgehog and vortex-like configurations, can be expressed in terms of a Lambert function whose argument depends explicitly on the magnetic parameters characterizing the magnetic skyrmion at each temperature. Unlike other formalisms based on 1D domain wall distributions, this was accomplished starting from a recently proposed 2D equilibrium magnetization that reduces to the Belavin–Poliakov solitonic solution in the isotropic case and for dominating exchange interaction. Exploiting the analogy between the behavior of particles in a gas and a population of skyrmions diameters, the Helmholtz free energy and the partition function were calculated treating the diameters population within the framework of a microcanonical ensemble. It was shown that, like for particles in an ideal gas, the skyrmions population is also characterized by a pressure strictly depending on the confining potential at each temperature, and in turn related to the exchange and magnetostatic interaction characterizing magnetic skyrmions. It was found that skyrmions pressure decreases monotonically with the volume but for volumes higher than the average volume an oscillatory behavior is superimposed onto the monotonic behavior because of the entropic contribution to the pressure that is one order of magnitude less than the average energy contribution. This trend occurs at each temperature and external field amplitude. An equation of state for a skyrmions population, regarded as the equivalent of the equation of state for an ideal gas, was derived and it was shown that it is valid for any type of magnetization texture. The equation of state reduces to that of an ideal gas when the skyrmion volume equals the average skyrmion volume at each temperature and external bias field.

These theoretical results on the thermodynamic properties of chiral magnetic skyrmions could pave the way for calorimetric measurements that allow determining the heat released or absorbed by the ferromagnetic system hosting the skyrmion and could enable the measurement of the free energy, exploiting the relations between heat exchanged and entropy, and heat exchanged, internal energy, and work done by the system. In this way, a direct measurement of the specific heat characterizing a population of skyrmions in a ferromagnetic system could also be carried out. These measurements would also enable verifying the predicted equation of state characterizing the statistical thermodynamic behavior of a skyrmions population and to experimentally determine the corresponding pressure. Finally, the analysis extended to very low temperatures would allow exploring quantum effects on the thermodynamic variables.

## Figures and Tables

**Table 1:** Calculated *a* and <*D*_sky_> used in the numerical calculations^39^.

*T* (K)	*a(×*10*^−5^* J*/*m*^2^)* *μ*_0_*H =* 0 mT	*<D*_sky_*> (*nm*)* *μ*_0_*H =* 0 mT	*a(×*10^−5^ J*/*m^2^*)* *μ*_0_*H =* 25 mT	*<D*_sky_*> (*nm*)* *μ*_0_*H =* 25 mT	*a(×*10^−5^ J*/*m*^2^)* *μ*_0_*H =* 50 mT	*<D*_sky_*> (*nm*)* *μ*_0_*H =* 50 mT
0	8.58	-	13.12	-	17.25	-
50	6.42	31.18	11.15	24.68	15.03	21.44
100	5.15	35.20	9.67	27.35	13.85	22.86
150	3.86	40.97	8.46	30.06	12.57	24.70
200	2.69	48.99	7.33	33.19	11.28	26.95
250	1.62	62.00	6.33	36.75	10.04	29.61
300	0.71	88.12	5.32	41.56	9.02	32.29

**Table 2:** Calculated *D*_0 sky_ used in the numerical calculations^39^.

*T* (K)	*D*_0 sky_* (*nm*)* *μ*_0_*H =* 0 mT	*D*_0 sky_* (*nm*)* *μ*_0_*H =* 25 mT	*D*_0 sky_* (*nm*)* *μ*_0_*H =* 50 mT
0	26.88	21.73	19.75
50	30.83	24.42	21.22
100	34.43	26.83	22.42
150	39.64	29.23	24.02
200	46.85	32.03	26.03
250	58.46	35.24	28.43
300	81.28	39.64	30.83

**Table 3:** Calculated values of *η* used in the numerical calculations.

*T* (K)	*η* *μ*_0_*H =* 0 mT	*η* *μ*_0_*H = *25 mT	*η* *μ*_0_*H = *50 mT
0	0.18	0.14	0.10
50	0.16	0.12	0.08
100	0.14	0.10	0.06
150	0.12	0.08	0.04
200	0.10	0.06	0.02
250	0.08	0.04	0
300	0.05	0.01	−0.02

## Appendix A

In this Appendix, some steps leading to the derivation of the different contributions to the total skyrmion energy are outlined.

In order to integrate the energy density of Equation (4) of the main text, the following trigonometric identities are considered:

(A1) $$\sin\left(2\arctan\left[\frac{B}{r}e^{-\;\xi r}\right]\right)\cos\left(2\arctan\left[\frac{B}{r}e^{\;-\;\xi r}\right]\right)=2B\frac{r\left(r^{2}e^{\;-\;\xi r}-B^{2}\;e^{-\;3\xi r}\right)}{{\left(r^{2}+B^{2}e^{-\;2\xi r}\right)}^{2}}$$

(A2) $$\sin^{2}\left(2\arctan\left[\frac{B}{r}e^{\;-\;\xi r}\right]\right)=4B^{2}\frac{r^{2}\;e^{-\;2\xi r}}{{\left(r^{2}+B^{2}e^{-\;2\xi r}\right)}^{2}}$$

(A3) $$\cos\left(2\arctan\left[\frac{B}{r}e^{-\;\xi r}\right]\right)=\frac{r^{2}-B^{2}e^{-\;2\xi r}}{r^{2}+B^{2}e^{-\;2\xi r}}$$

(A4) $$\cos^{2}\left(2\arctan\left[\frac{B}{r}e^{-\xi r}\right]\right)={\left(\frac{r^{2}-B^{2}e^{\;-2\xi \;r}}{r^{2}+B^{2}e^{\;-2\xi \;r}}\right)}^{2}.$$

Substituting Equations (A1)–(A4) into Equation (4) and performing some trivial algebraic manipulations, one gets:

(A5) $$\begin{array}{l} \varepsilon_{\mathrm{tot}}\left(r,r_{\mathrm{sky}}\right)=4\tilde{A}\;B^{2}\left[\frac{\;\left(2+2r\xi +r^{2}\xi^{2}\right)e^{-2\xi r}}{{\left(r^{2}+B^{2}e^{-2\xi r}\right)}^{2}}\right]\pm 2\tilde{D}\;B\left[\pm \frac{\left(r^{2}-B^{2}\;e^{-2\xi r}\right)e^{\;-\xi r}}{{\left(r^{2}+B^{2}e^{-2\xi r}\right)}^{2}}\mp \frac{(1+r\xi )e^{-\xi r}}{r^{2}+B^{2}e^{-2\xi r}}\right]\\ +K_{u}-K_{\mathrm{eff}}{\left(\frac{r^{2}-B^{2}e^{-2\xi r}}{r^{2}+B^{2}e^{-2\xi r}}\right)}^{2}\mp \mu_{0}M_{s}H_{\mathrm{ext}}\frac{r^{2}-B^{2}e^{-2\xi r}}{r^{2}+B^{2}e^{-2\xi r}}. \end{array}$$

Equation (A5) is Equation (5) of the main text. The integrals appearing in the compact expression of the energy given in Equation are listed the following. The integrals for the exchange contribution are:

(A6) $$I_{1\text{-}\mathrm{exch}}=2B^{2}\int_{0}^{r_{d}}\frac{r\;e^{-2\xi r}\;}{{\left(r^{2}+B^{2}e^{-2\xi r}\right)}^{2}}\;dr$$

(A7) $$I_{2\text{-}\mathrm{exch}}=2B^{2}\xi \int_{0}^{r_{d}}\frac{\;r^{2}\;e^{-2\xi r}\;}{{\left(r^{2}+B^{2}e^{-2\xi r}\right)}^{2}}\;dr$$

(A8) $$I_{3\text{-}\mathrm{exch}}={\left(B\xi \right)}^{2}\int_{0}^{r_{d}}\frac{\;r^{3}\;e^{-2\xi r}\;}{{\left(r^{2}+B^{2}e^{-2\xi r}\right)}^{2}}\;dr.$$

Instead, the integrals for the DMI (either IDMI or bulk DMI) are:

(A9) $$I_{1\text{-DMI}}=\pm B\int_{0}^{r_{d}}\frac{r^{3}e^{-\xi \;r}\;}{{\left(r^{2}+B^{2}e^{-2\xi \;r}\right)}^{2}}dr$$

(A10) $$I_{2\text{-DMI}}=\mp B^{3}\int_{0}^{r_{d}}\frac{r\;e^{-3\xi r}\;}{{\left(r^{2}+B^{2}e^{-\xi r}\right)}^{2}}dr$$

(A11) $$I_{3\text{-DMI}}=\mp B\int_{0}^{r_{d}}\frac{r\;e^{-\xi \;r}\;}{r^{2}+B^{2}e^{-2\xi \;r}}dr$$

(A12) $$I_{{}_{4\text{-DMI}}}=\mp B\xi \int_{0}^{r_{d}}\frac{r^{2}e^{-\xi r}\;}{r^{2}+B^{2}e^{-2\xi \;r}}dr.$$

The integral for the effective anisotropy reads:

(A13) $$I_{\mathrm{ani}}^{\mathrm{eff}}=\int_{0}^{r_{d}}\frac{{\left(r^{2}-B^{2}e^{-2\xi r}\right)}^{2}\;}{{\left(r^{2}+B^{2}e^{-2\xi r}\right)}^{2}}rdr.$$

Finally, the integral associated to the external bias field is:

(A14) $$I_{\mathrm{extfield}}^{}=\int_{0}^{r_{d}}\frac{r^{2}-B^{2}e^{-2\xi r}\;}{r^{2}+B^{2}e^{-2\xi r}}rdr.$$
